# Slavs in the closet: computational genomic analysis reveals cryptic slavic signatures in the Avar Khaganate and their contribution to medieval Croatian population formation

**DOI:** 10.3389/fgene.2025.1610942

**Published:** 2025-09-22

**Authors:** Todor Chobanov, Svetoslav Stamov, for Bulgarian Academy of Science and Institute for Balkan Studies

**Affiliations:** ^1^ Institute for Balkan Studies, Bulgarian Academy of Science, Sofia, Bulgaria; ^2^ Center for Interdisciplinary Research and Paleogenomic Knowledge, National History Museum, Sofia, Bulgaria

**Keywords:** ancient DNA, Slavic migrations, Avar Khaganate, Croatian ethnogenesis, computational genomics, principal component analysis, population genetics

## Abstract

Our study applies a systematic computational genomic approach to investigate the complex population dynamics of Southern Slavs in the Hungarian Plain and Avar Khaganate, and their subsequent role in forming the medieval Croatian population. Using a quality-controlled dataset of 1,800 ancient DNA samples, we implemented a comprehensive analytical framework centered on systematic screening of marginal Principal Components to detect cryptic Slavic genetic signatures. This strategic methodological approach addresses the well-documented analytical challenge that Germanic and Slavic populations remain indistinguishable using conventional PC1-2 analysis due to shared Baltic Bronze Age ancestry. Through systematic evaluation of all principal components (PC1-20), we identified PC9 as a reliable indicator of Slavic ancestry within European ancient DNA samples when combined with PC4 and PC3. This approach revealed substantial Baltic genetic components in early Slavic populations (57% in Slovakia/Slovenia) decreasing to 39%–51% in medieval Croatian samples. Statistical modeling demonstrates that contemporary Croatian populations formed through three distinct migration waves, with 50%–60% total Slavic ancestry and 20%–25% pre-Slavic Balkan continuity. Significantly, we identified individuals with Slavic genetic profiles in prestigious Avar burial contexts, questioning established understanding of social hierarchies within the Khaganate. The genomic evidence indicates that key aspects of South Slavic genetic structure emerged through interactions within the Carpathian Basin rather than after Balkan arrival. Our findings demonstrate that Croatian ethnogenesis involved gradual integration rather than population replacement, with the Avar Khaganate serving as a crucial demographic interface where South Slavic genetic structure emerged. Our approach addresses longstanding historical questions regarding Croatian ethnogenesis by identifying specific genetic signatures and quantifying their population-level contributions, demonstrating how application of computational genomics provides unprecedented resolution in studying complex population transformations when traditional historical and archaeological approaches reach interpretive limits.

## 1 Introduction

The Great migration period (AD300–600) saw the transformation of “the Balkans” in political, cultural, and demographic sense. One of the most intriguing questions is how the various modern peoples inhabiting the region in recent times started their history in the aftermath of the Great Migration and the beginning of the Early Medieval era. Considering the modern states and the existing ethnicities, we have been engaged in the last three centuries in numerous attempts to trace their beginning–with mixed and sometimes controversial results. This has been particularly challenging in the case of the various Slavic speaking communities, that apparently belong to one broad and continuous wave of similar tribes that settled in the region during the period in question. The present article tries to better approach the beginning of Croatian history on the Balkans by combining the traditional written and archaeological sources with genomic data, derived from the ever-growing available database of ancient DNA.

The earliest preserved written source, labelling the Croats as a separate polity (and respectfully–clearly distinguishable ethnicity) originates from the late 9th c., which is about three centuries after their likely arrival in the lands of the Eastern Roman empire. It is a commemorative dedication inscription, excavated in a modest single nave church, mentioning “Trepimirus, dux Chroatorum,” who ruled over the “regnum Chroatorum”. The inscription has brought some serious discussion during the decades after its discovery and is presently accepted as the earliest certain mentioning of Croats and their state (Djino, 2010). Far more informative, but also self-contradictory, are the reports provided in the 10th century treatise *De Administrando imperio*, composed under the guidance of emperor Constantine VII Porphyrogenitus. Intriguingly, the arrival of the Croats is mentioned not once as with most other described peoples, but three times–in chapters 29, 30 and 31. The three reports contain similarities, but also contradictions, a situation that could be explained if we agree that the emperor ordered different assistants with the composition of different parts of the treatise. D. Djino’s explanation that the stories in chapters 29 and 30 represent recorded folk-tales - the Dalmatian tale of the fall of Salona and the Croat origo gentis, where the content of chapter 31 represents an attempt to combine them, also sounds plausible (Djino, 2010).

Chronology-wise the most interesting data comes from the content of the abovementioned chapter 31, specifically devoted to the history of Croatians. It claims that they arrived from their homeland “beyond Turkey (Hungary) and next to Francia (the Frankish kingdom)” in the times of emperor Heraclius seeking refuge and protection from Byzantium (Constantine Porphyrogenitus, 1967). Then, “*by command of the emperor Heraclius these same Croats defeated and expelled the Avars from those parts, and by mandate of Heraclius the emperor they settled down in that same country of the Avars, where they now dwell*” (Constantine Porphyrogenitus, 1967). An interesting detail in this version of the story is the name of chieftain of the Croats during the latter part of the described events–Porgas, which is none of the names listed in chapter 30, where the leaders of the Croats, representing a family, are called “*Kloukas and Lobelos and Kosentzis and Mouchlo and Chrobatos, and two sisters, Touga and Bouga”.* Chapter 30 also gives a slightly different version for the original homeland, reporting that it is „beyond Bavaria, where the Belocroats are now” (Constantine Porphyrogenitus, 1967). The non-Slavic sounding of most of those names, quite different from those of non-legendary figures like the 9th c. Trepimirus (Trpmir) may indicate that the those accounts developed in non-Slavic surroundings, or that the early Croat confederation of tribes may have been led by non-Slavic leaders during their exodus from Avar-controlled lands. Another implication from the report is that the Croats split from a wider group that did not disappear after their march to new lands (Istvánovits, 1998; Istvánovits, 2020; Kulcsár and Istvánovits, 2020).

The Avars represent the most solid and unchanging element in all the three versions and stories reported by Constantine VII–undoubtedly because the peak of Slavic massive invasions on the Balkans should be placed within the global context of Avar-Byzantine confrontation and later - the shrinking Avar control over the North-Western Balkans (after the Great siege of 626). As W. Pohl correctly acknowledged it in his capital work about the Avars that the Slavs on the Balkans faced a unique situation and were able to advance deep within Byzantine territories and later settle there, unlike their relatives in the West who were greatly limited in their military endeavors (Pohl, 2018). We cannot imagine the early Slavic advancements without the Steppe powers attacking the empire–first the Bulgars who took the mantle of the Huns from late 5th c., then the Avars, who represented a major threat due to the fact that they were resettling to a new homeland and strongly influenced the agenda of most of the tribes their encountered during their march to Panonia. Those historical developments are surely well reflected, even if distorted, in Constantine VII’s work.

The archaeological evidence is even less clear than the written record. The problem with the tracing of early Croatian presence in the field is similar to the challenge to trace any other early Slavic presence on the Balkans, including the Slavic tribes that would later participate in the formation of Danube Bulgaria Chobanov (2021) who’s habitation areas are much clearer and well reported in Byzantine sources. After observing all the available scientific data, W. Pohl concluded that *the relative lack of traces of the early Slavic population in many regions constitutes a major methodological problem* Pohl (2018). The material culture of those early Slavic groups, arriving to the Balkans from the same areas, was certainly similar and it is likely impossible to distinguish between the tribal groups based on artifacts in burials or the predominant rite–cremation. Another issue is that their small cemeteries are difficult to locate on the terrain in modern times, 1,400 years after the start of their migration to the Balkans. D. Djino, in his complex work, acknowledges the problem that the so-called “Old Croat” cemeteries that should likely be dated not in late 7^th^ – 8^th^ c., but later–after the end of the Avar khaganate in the last years of the 8^th^ c (Djino, 2010).

Apparently, possible solutions of this stale mate could come not from historical or archaeological record itself, but from a quite different perspective–the study of ancient DNA (ADNA), that in the last several years has become increasingly important for archaeologists around the word. During the last five-six years we witnessed significant accumulation of published ADNA samples, collected from present day Croatian lands, but also from areas where supposedly the Slavic migration towards the Balkans launched. The emergence of genome-wide studies enabled researchers to analyze hundreds of individuals across different time periods of Croatia itself. In the first study featuring samples from Croatian lands Mathieson et al. (2018) analyzed 225 individuals from southeastern Europe, establishing the baseline for understanding the genetic structure of early populations in the region. Spanning 12,000 to 500 BCE. The study documented the genetic continuity and admixture between early farmers from Anatolia and smaller groups of local hunter-gatherers and found that Croatian populations during the Neolithic and Bronze Age were influenced mostly by Anatolian farmers and later steppe (Yamnaya) migrations, both contributing to the region’s genetic diversity. Next study featuring Croatian samples was „Genome-wide analysis of nearly all the victims of a 6200-year-old massacre” by Novak et al. (2021), and contained genomic analysis of 38 samples, victims of a violenth deat and burried in a mass-grave. Results provided insights into the social and demographic structure of early farming communities in Croatia. Also in 2021, (Freilich et al., 2021) published at Nature Scientific Reports a study featuring genome-wide data from 28 individuals in eastern Croatia, spanning from the Middle Neolithic to Roman times. It revealed strong genetic continuity during the Neolithic and partial population replacement with evidence of first-cousin mating practices and patrilocal social organization in the Bronze Age. The study also identified unexpected hunter-gatherer-related ancestry in some Bronze Age communities, suggesting contacts with Baltic and northern Carpathian Basin populations. („Reconstructing genetic histories and social organisation in Neolithic and Bronze Age Croatia”).

A landmark study by Lazaridis (2022) analyzed 727 individuals across the “Southern Arc” region, positioning Croatian lands within a broader geographical and temporal context. Their research demonstrated that the region served as a crucial genetic bridge between West Asia and Europe, documenting significant population movements, including the influence of Yamnaya pastoralists from the Eurasian steppe during the Bronze Age. It was the first study to examine the impact of Slavic migrations during the early medieval period and how it reshaped the genetic landscape of the Balkans, and in Croatian lands in particular. The findings from the study confirmed that Croatian populations were shaped by multiple waves of migration and admixture: Early Anatolian farmers during the Neolithic; Steppe pastoralists during the Bronze Age; Roman and Byzantine influences during antiquity and Slavic migrations during the medieval period (Gnecchi-Ruscone et al., 2021; Gnecchi-Ruscone et al., 2022) and (Lehti et al., 2021). The study concluded that significant genetic shifts occurred due to Roman colonization and later - Slavic migrations, completely reshaping the genetic landscape of medieval Croatia.

Despite the advances, a critical methodological challenge emrged, limiting our ability to trace Slavic population movements. Early Germanic and early Slavic populations proved difficult to distinguish using conventional principal component analysis. Both groups share substantial Baltic Bronze Age (McColl, 2024) ancestry, which causes PC1-2 analysis to place early Germanic and early Slavic individuals in mixed order without clear separation. This limitation forced Olalde et al. to employ marginal reference populations, such as Russia_Iron Age Ingria rather than obvious Slavic references, when modeling Balkan Slavic populations (Olalde et al., 2023). The persistence of this challenge has hindered detailed reconstruction of the specific mechanisms through which Slavic populations contributed to medieval Croatian ethnogenesis.

Recent research increasingly focused on the genetic impact of the groups from the Great Migrations period on the populations from Croatian lands. Olalde et al. (2023) examined the genetic changes in Croatia during the Roman period and subsequent Slavic migrations. The study revealed that while Roman cultural influence was substantial, the genetic impact of Italic populations was relatively limited-to-entirely absent. Instead, they identified significant genetic contributions from Anatolian and, to a lesser extent, North African populations during the Roman period. The study identified individuals with Central/Northern European and Pontic-Kazakh Steppe ancestry, reflecting the arrival of diverse “barbarian” groups (e.g., Goths, Huns) during the Migration Period. Olalde’s research illuminated the crucial character of Slavic migrations. Resuts demonstrated that Croatian lands withnessed one of the most significant demographic changes in European history during Early MA (600–800 CE), with Slavic migrations contributing between 30%–60% of the ancestry to Balkan populations, with effect maximized on the populations from contemporary Croatian lands (60%). These migrations involved both male and female individuals, resulting in a more balanced demographic impact compared to earlier population movements. The cumulative evidence from these studies demonstrated that the genetic makeup of contemporary Croatian populations reflects contributions from early Anatolian farmers, Bronze Age steppe populations, Roman-era Anatolian migrants, but most significantly, Slavic populations, creating a unique genetic profile that persists in modern Croatian populations.

While recent studies have advanced our understanding of Croatian population history, several areas remain to be fully explored. These include the precise timing and nature of Slavic admixture with local populations, the relationship between registered by ADNA studies genetic changes and documented by historical research historical events and the impact of smaller-scale population movements during the medieval period. These gaps in our knowledge present opportunities for future research to further illuminate the population history of Croatian lands. The present study aims to narrow the gap between macro-level genetic analyses conducted so far and micro-level population dynamics in the context of our historical knowledge about early medieval Croatian population formation. While previous ADNA research had established broad patterns of population movement and admixture in the Balkans, significant questions remain about the specific mechanisms of population formation, number of slavic migrations and the role of Avar Khaganate (Maenchen-Helfen, 1973) in the peopleing of the former Roman territories (Mócsy, 1974) during Early MA.

## 2 Research objectives and analytical strategy

**FIGURE 1:**
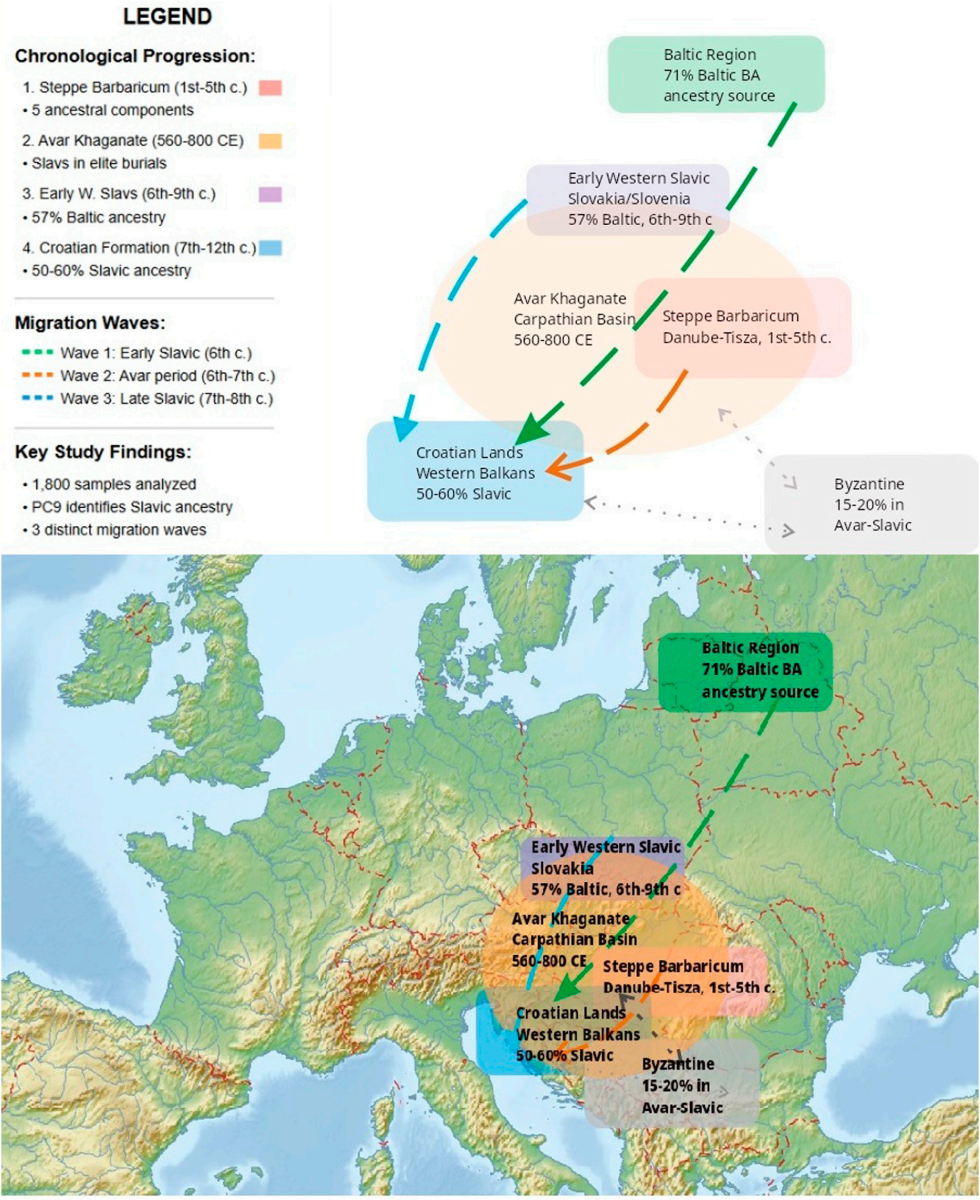
Geographical Context Map. Geographic and chronological context of the study area. The map shows key regions involved in Slavic population movements and Croatian ethnogenesis (300–1,200 CE): Baltic Region (source of Baltic Bronze Age ancestry, 71% in modern Lithuanians), Early Western Slavic territories in Slovakia/Slovenia (57% Baltic ancestry, 6th-ninth century CE), Avar Khaganate in the Carpathian Basin (560–800 CE), Steppe Barbaricum between Danube and Tisza rivers (1st-fifth century CE), and Croatian lands in the Western Balkans (50%–60% Slavic ancestry). Three migration waves are indicated by arrows: Wave 1 (Early Slavic, 6th century), Wave 2 (Avar period, 6th-7th century), and Wave 3 (Late Slavic, 7th-8th century). The Byzantine sphere of influence (15%–20% genetic contribution to Avar-Slavic populations) is also shown.

The present study systematically addresses methodological limitations in ancient DNA population analysis while investigating fundamental questions about Croatian population formation that remain incompletely resolved. The geographic scope encompasses key regions of Central and Southeast Europe: the Baltic region (modern Lithuania and Latvia), the Carpathian Basin including the territories of the Avar Khaganate (modern Hungary), the Steppe Barbaricum flatlands between the Danube and Tisza rivers, and the Western Balkans including medieval Croatian lands (Figure 1).

We sought to determine: (1) the precise timing and mechanisms of Slavic admixture with local Balkan populations during the 6th-9th centuries CE; (2) the number and genetic signatures of distinct Slavic migration waves; (3) the role of the Avar Khaganate (560–800 CE) in facilitating Slavic population movements; and (4) quantitative relationships between genetic changes documented through ancient DNA analysis and historical events recorded in contemporary sources (Vernadsky, 1959), particularly Constantine VII Porphyrogenitus’s De Administrando Imperio.

By analyzing 1,800 quality-controlled ancient DNA samples from these key regions spanning 100–1,200 CE, we aimed to provide unprecedented resolution in studying the complex population transformations that shaped medieval Croatian ethnogenesis. Our approach centers on systematic evaluation of all principal components (PC1-20) rather than conventional focus on PC1-2, which fails to distinguish Germanic from Slavic populations due to shared Baltic Bronze Age ancestry (McColl, 2024). This systematic screening, combined with formal population genetic modeling using qpAdm and qpWave, enables detection of cryptic Slavic genetic signatures in archaeological contexts where cultural attribution remains ambiguous.

## 3 Methods

### 3.1 Dataset assembly and sample selection

**FIGURE 2:**
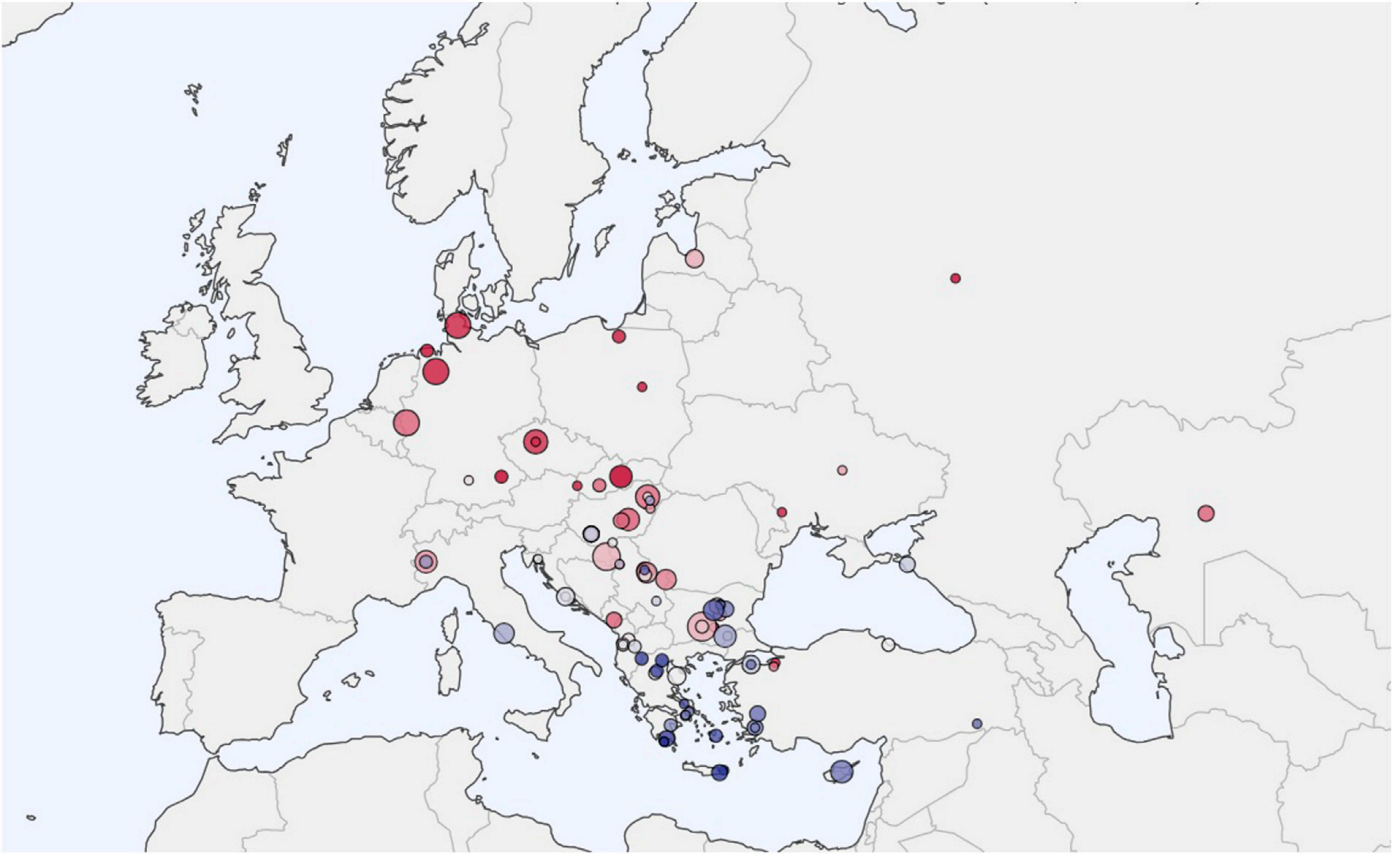
Sample Locations Map. Distribution of 1,800 ancient DNA samples analyzed in this study. Samples span from the Baltic region to the Balkans and from Western Europe to the Pontic Steppe, covering the period 500 BCE-1200 CE. Larger circles indicate sites with multiple individuals. Red markers indicate Slavic and proto-Slavic populations, blue markers indicate reference populations, and gray markers indicate comparative populations. Sample density is highest in the Carpathian Basin and Western Balkans, reflecting the focus on Avar Khaganate and Croatian ethnogenesis.

We assembled a comprehensive ancient DNA dataset from the Allen Ancient DNA Resource (AADR) maintained by the David Reich Laboratory at Harvard Medical School (version 54.1, released January 2024). The initial collection comprised 3,600 ancient DNA samples from Eurasia spanning 800 BCE to 1,200 CE. The geographic distribution of our 1,800 samples is illustrated in Figure 2, extending from the Baltic region to the Balkans and covering the period from 500 BCE to 1200 CE.

Our regional representation imcluded 400 samples from the Hungarian Plain and Carpathian Basin dating from 300 BCE–900 CE, 185 samples from the Balkan Peninsula spanning 700 BCE–1,200 CE, and 133 samples from Central and Eastern Europe covering 300 BCE-900 CE. Additionally, we included 131 samples from Baltic and Scandinavian regions dating to 300 BCE-1,100 CE, and 44 samples from Western Steppe and Pontic regions from 400 BCE–800 CE. Sample selection prioritized individuals from archaeologically well-contextualized sites with clear cultural attribution and radiocarbon dating, with complete supporting metadata including site locations, archaeological context, and dating information. We provide full list of samples we used in the supplement.

### 3.2 Quality control and filtering procedures

Quality control procedures adhered to established protocols for ancient DNA analysis (Patterson et al., 2012; Maier and Reich, 2023).

We implemented stringent filtering criteria at both SNP and individual levels. For SNP-level filtering, we retained only those variants with minor allele frequency of at least 0.01, Hardy-Weinberg equilibrium p-values exceeding 10^–6^, and maximum missing genotype rates not exceeding 80%. We further removed SNPs in linkage disequilibrium using an *r* ^2^ threshold of 0.25, with 200 SNP windows and 50 SNP steps.

At the individual level, we required minimum coverage of 50,000 autosomal SNPs and maximum missing genotype rates of 80% and we verified consistency between genetic and archaeological sex determination. To avoid pseudoreplication, we performed kinship filtering to remove first-degree relatives identified by PI_HAT values exceeding 0.25. Following these quality control procedures, our final analytical dataset comprised 1,800 individuals genotyped at 540,000 autosomal SNPs.

### 3.3 Principal component analysis and systematic screening

Principal Component Analysis has been extensively applied in population genetics since Cavalli-Sforza (1994), with traditional approaches emphasizing the first two or three principal components that capture the greatest genetic variance.

We performed principal component analysis using PLINK 2.0 (alpha 3.7). We first computed standard PC1-2 to confirm the documented Germanic-Slavic discrimination problem, as shown in Supplementary Figure S1. We then implemented our systematic screening protocol through a four-stage process. First, we generated 20 principal components from the filtered genotype matrix for comprehensive PC calculation. Second, we systematically evaluated each PC to identify populations showing extreme values exceeding two standard deviations from the mean. Third, we assessed each PC’s capacity to separate early Slavic from Baltic and Germanic populations through discrimination testing. Finally, we tested all possible three-way PC combinations to identify the optimal combination for maximum population discrimination. The complete screening protocol is available at github.com/StamovS/slavic-signatures-avar/scripts/pc_screening_analysis.R.

### 3.4 Population genetic modeling

Formal admixture tests and ancestry proportion estimation employed qpAdm and qpWave implemented in AdmixTools 2.0.1 (Harney et al., 2021). Our reference population framework consisted of primary references including CEE_EarlyMedieval representing Early Western Slavic populations and Latvia_BA representing Baltic Bronze Age. Secondary references encompassed Hungary_Avar populations totaling 256 individuals, Balkans_IA with 89 individuals, and Byzantine populations comprising 112 individuals. The outgroup panel included Mbuti, Han, Karitiana, Papuan, Onge, Iran_N, WHG, and MA1 to ensure robust model fitting.

Our model selection criteria involved testing between two and five source population models, requiring p-values exceeding 0.05 for acceptable model fit. We used qpWave to determine the minimum number of sources needed for adequate modeling and validated results using block bootstrap with 1,000 replicates. All qpAdm configuration files and parameters are available at github.com/StamovS/slavic-signatures-avar/qpadm_configs/.

### 3.5 Statistical analysis

For Euclidean distance calculations in PC space, we computed genetic distances using the formula d (i,j) = √(Σ_k=1_^3^ (PC_ki_ - PC_kj_)^2^), where PC_ki_ represents the *k*th principal component score for individual i, using optimal PC combinations, typically PC9, PC4, and PC3. For cluster analysis, we employed Ward’s method on Euclidean distance matrices for hierarchical clustering, while multidimensional scaling used classical metric MDS implemented in R.

### 3.6 Computational implementation

All analyses were implemented in R version 4.3.1, utilizing PLINK 2.0 for genotype processing and PCA, AdmixTools 2.0.1 for formal admixture modeling, and tidyverse 2.0.0 with ggplot2 3.4.2 for data manipulation and visualization. We developed custom R scripts for systematic PC screening and population analysis. The complete analytical pipeline comprising five integrated modules is available at github. com/StamovS/slavic-signatures-avar with comprehensive documentation, error handling, and example datasets enabling full reproducibility.

## 4 Results

### 4.1 Principal component analysis reveals cryptic slavic signatures

Standard principal component analysis using PC1-2 (Supplementary Figure S1) confirmed the well-documented challenge of distinguishing Germanic from Slavic populations due to shared Baltic Bronze Age ancestry. These primary components, which capture the greatest genetic variance, place early Germanic and early Slavic individuals in overlapping clusters without clear separation, forcing previous studies to employ unexpected reference populations when modeling Balkan Slavic ancestry (Olalde et al., 2023).

Our systematic screening of all twenty principal components identified PC9 as capturing a distinctive ancestry signal that effectively discriminates early Slavic populations within European ancient DNA samples (Supplementary Table S1). While PC9 reaches maximum values globally in northern Eurasian populations including Nganasan (0.0567), Ket, and European Saami, within European samples this component consistently maximizes in early Western Slavic populations from Slovakia and Slovenia dating to the 6th-9th centuries CE.

**FIGURE 3:**
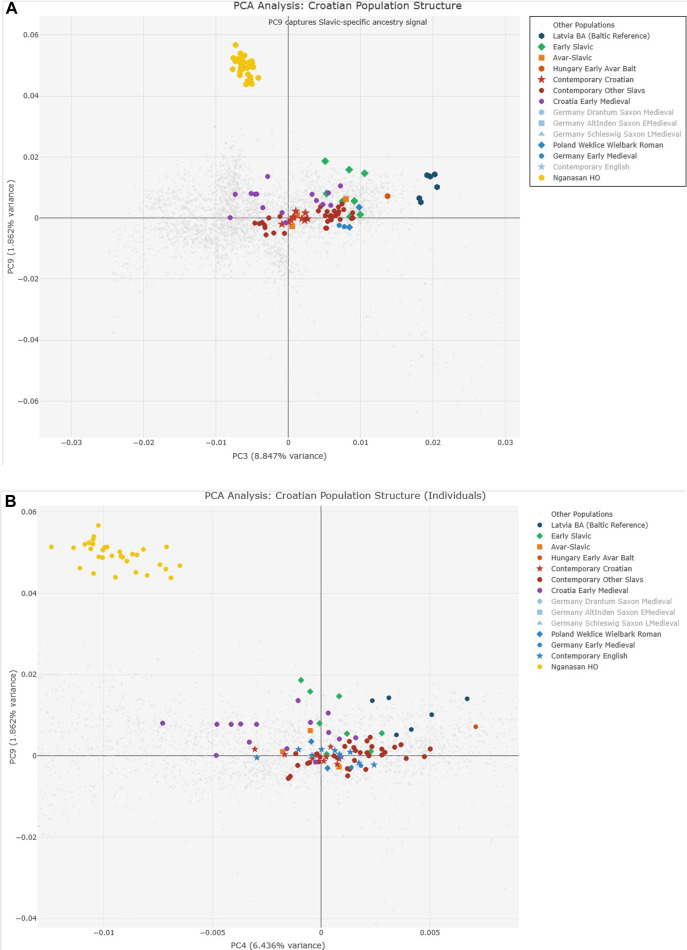
Principal Component Analysis of 1,800 ancient DNA samples. **(A)** PC3 vs. PC9 showing Slavic-specific ancestry signal with Early Slavic populations (green) displaying highest PC9 values. **(B)** PC4 vs. PC9 providing optimal discrimination between Early Slavic, Avar-Slavic (orange), and Contemporary Croatian (red) populations. Table insert (Supplementary Table S1) shows PC9 extreme values identifying CEE_EarlyMedieval as having highest European PC9 values (0.0186). The systematic decrease in PC9 from Early Slavic through Contemporary populations tracks progressive admixture.

Analysis of PC9 extremes (Supplementary Table S1) revealed CEE_EarlyMedieval samples show the highest European values. The systematic screening approach identified PC9 combined with PC3 and PC4 as optimal for Slavic ancestry detection (Figures 3A,B).

Analysis of PC9 extremes (Supplementary Table S1) revealed that CEE_EarlyMedieval samples show the highest European values (0.0186), followed by various Croatian medieval samples (Croatia_Brekinjova: 0.0162, Croatia_Jagodnjak: 0.0158, Croatia_Sibeník: 0.0153). The presence of Latvia_BA samples with elevated PC9 values (0.0143–0.0128) confirms the Baltic Bronze Age connection to early Slavic populations. The systematic screening approach identified PC9 combined with PC3 and PC4 as optimal for Slavic ancestry detection (Figures 3A,B).

The heat map analysis (Supplementary Figure S2) of genetic distances in PC9,4,3 space demonstrates clear population clustering that is absent in conventional PC1-2 analysis. Hungarian Avar-period Slavic samples (Hungary_EarlyAvarslav, Hungary_LateAvarslav) cluster tightly with Croatian medieval samples, while maintaining distinct separation from Germanic and other non-Slavic populations.

### 4.2 Validation of slavic ancestry detection method

Our methodology successfully identified samples previously classified as Slavic by independent research teams, validating the systematic PC screening approach. These validation samples included Byzantine_oEuropean outlier samples) identified by Reich and Lazaridis as “Slavic admixed,” Czech_Medieval samples, displaying expected temporal and geographic patterns, Hungary_Avar context samples AV1 and AV5 independently identified as early Slavic individuals, and Croatia_Medieval_o samples considered by the Reich team as representing early Slavic presence.

The discriminating power of PC9 appears to capture an ancient Paleolithic Siberian ancestry component that was absorbed differentially by various Bronze Age populations. This component is absent in Mesolithic European hunter-gatherers but present in Bronze Age populations from the Sintashta-Alakul complex (2,100–1,800 BCE) and Kazakhstan MLBA cultures (1800–1500 BCE), suggesting its introduction through steppe-mediated gene flow.

### 4.3 Pre-Migration population structure in the steppe barbaricum (1st-5th century CE)

Analysis of thirty individuals from the Steppe Barbaricum, the 150-km flatland strip between the Danube and Tisza rivers, revealed extraordinary genetic complexity that predates traditional accounts of Slavic presence by several centuries.

The population composition shows remarkable balance among five ancestral components (Supplementary Figure S3): Germanic (21.5%), Balkan Iron Age (20.8%), Proto-Slavic (20.2%), Byzantine (19.1%), and Steppe Sarmatian-Hunnic (16.9%). This balanced distribution, confirmed through qpAdm modeling with high statistical confidence (p = 0.73), suggests extensive intermarriage and cultural exchange rather than segregated ethnic communities.

Significantly, Proto-Slavic signatures absent in second-century samples emerge clearly in 5th-century individuals, suggesting Slavic presence in the Hungarian Plain by at least 400 CE—over a century before historical documentation. Temporal analysis reveals progressive increase in Proto-Slavic components from 0% (2nd century) to 20.2% (5th century), while Sarmatian components decrease from 35% to 16.9% over the same period.

This admixed Barbaricum population contributed substantially to later groups: 20%–40% to Early Western Slavic samples and approximately 20% to medieval Croatian populations. The linguistic evidence, including the possibly Slavic term “strava” recorded at Attila’s court (453 CE) and the ethnonym “Limigantes” (meaning “speakers”), gains new significance in light of these genetic findings.

### 4.4 Slavic presence in the Avar Khaganate (560–800 CE)

Comprehensive analysis of nearly 300 individuals from Avar-period cemeteries (Supplementary Figure S5) in the Carpathian Basin revealed patterns challenging traditional narratives of strict ethnic hierarchies within the Khaganate.

Twelve individuals with predominantly Slavic genetic profiles (>50% Early Western Slavic ancestry) were identified in prestigious burial contexts, including graves with weapons, horse sacrifices, and high-status ornaments. Notable examples include SZOD1-829 from the 6th-7th century with 67% Slavic ancestry, buried with sword and belt set; OBT-56 from the 8th century with 72% Slavic ancestry in an elite warrior burial with horse; and Sample Av5 from 560 CE, representing the earliest confirmed Slavic individual in an Avar context.

The multidimensional scaling plot (Supplementary Figure S5) shows clear separation between different Avar-period populations. Hungary_EarlyAvarBalt clusters near Latvia_BA, confirming the presence of individuals with predominantly Baltic ancestry (59%–75%, Supplementary Figure S7) within the Khaganate. These likely represent either direct Baltic migrants or preserved unadmixed early Slavic populations.

Avar-Slavic genetic profiles (Supplementary Figure S8) from the late period (700–800 CE) show distinct composition patterns, incuding substantial Byzantine components (15%–20%), indicating ongoing interaction with imperial territories. The composition—Early Western Slavic (59.2%), Roman-Byzantine (20.9%), and Steppe Post-Hunnic (19.9%)—demonstrates that Slavic populations within the Khaganate maintained distinct genetic identity while incorporating regional elements.

### 4.5 Baltic ancestry gradient documents migration routes

Euclidean distance analysis in PC9, 4, 3 space (Supplementary Figure S6) reveals clear genetic relationships between Baltic and Central/Eastern European populations. Plotting the distance to the nearest Latvia_BA sample against the distance to the nearest CEE_EarlyMedieval sample showed that Croatian individuals form a continuous genetic gradient linking Baltic references to the Balkan populations from antiquity. Early Slavic individuals cluster tightly near the origin, reflecting their dual genetic affinity to both Baltic Bronze Age and Early Western Slavic populations. In contrast, present-day Slavic groups appear more dispersed, indicating additional post-migration admixture. Croatian samples (green) form a continuous gradient between Baltic reference and other European populations, supporting the multi-wave migration model.

**FIGURE 4:**
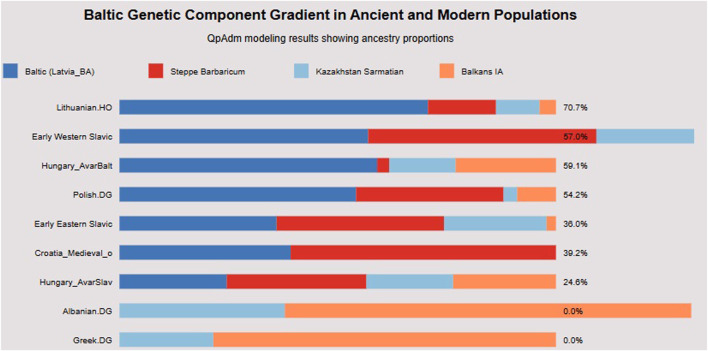
Baltic ancestry gradient. This horizontal bar chart quantifies Baltic Bronze Age ancestry proportions across populations using qpAdm modeling. Lithuanian. HO retains the highest Baltic ancestry (70.7%), establishing the maximum for modern populations. The gradient demonstrates clear geographic and temporal patterns: Early Western Slavic (57% Baltic), Hungary_AvarBalt (59.1%), and Polish. DG (54.2%) maintain substantial Baltic components, while Croatia_Medieval_o shows intermediate levels (39.2%), and Hungary_AvarSlav shows further dilution (24.6%). Crucially, Albanian. DG and Greek. DG show 0% Baltic ancestry, confirming that this component specifically tracks Slavic rather than general European ancestry. The presence of Steppe Barbaricum, Kazakhstan Sarmatian, and Balkans IA components in varying proportions demonstrates that Slavic populations formed through admixture with local populations encountered during migration. This gradient provides quantitative support for the Baltic homeland hypothesis and demonstrates that Croatian populations retain approximately 39%–51% of the original Baltic genetic signature, consistent with substantial but not complete population replacement during medieval formation.

Formal qpAdm modeling revealed a clear gradient of Baltic Bronze Age ancestry (Figure 4) that traces Slavic migration routes from the Baltic homeland to the Balkans.

Modern populations show decreasing Baltic ancestry with geographic distance from the Baltic region. Lithuanian. HO retains 71% Baltic Bronze Age ancestry, while Polish. DG shows 54.2%. Early Western Slavic populations from Slovakia and Slovenia maintain 57%, and the Hungary_AvarBalt individual (Supplementary Figure S7) shows 59%–75%. Croatian populations demonstrate intermediate levels, with Croatia_Medieval_o showing 39%–51% and Hungary_AvarSlav showing 24.6%.

Critically, non-Slavic Balkan populations show no significant Baltic component, with Albanian. DG, Greek. DG, and Balkans_IA all showing 0% Baltic ancestry. This gradient provides quantitative support for the Baltic origins hypothesis and demonstrates that Croatian populations retain 39%–51% of the original Baltic genetic signature, consistent with substantial but incomplete population replacement during medieval formation.

### 4.6 Multi-wave migration model for Croatian ethnogenesis

**FIGURE 5:**
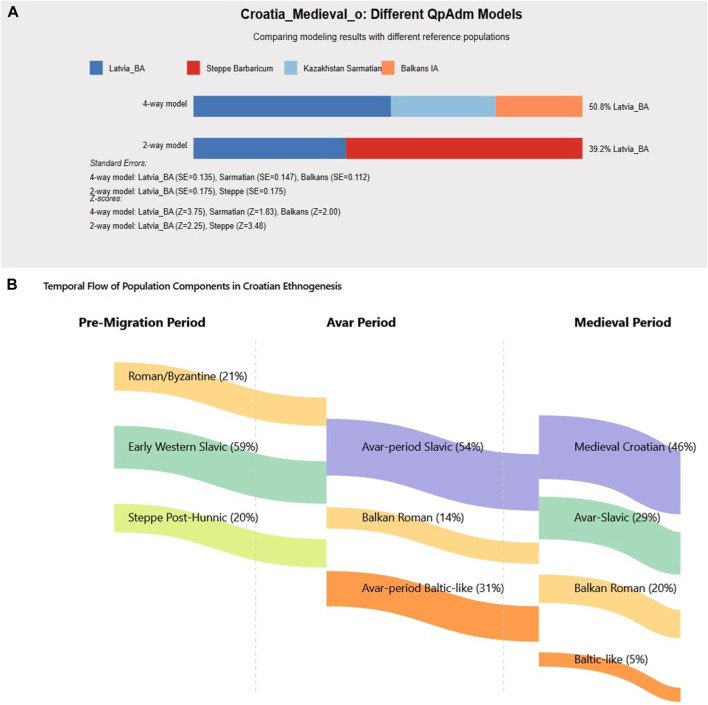
Medieval Croatian Population Formation Models. Multi-wave migration model for Croatian ethnogenesis. **(A)** Croatia_Medieval_o admixture showing 50.8% Baltic ancestry in earliest samples. **(B)** Temporal flow diagram demonstrating population component transformations from Pre-Migration through Avar Period to Medieval Croatian formation.

Statistical modeling demonstrates contemporary Croatian populations formed through at least three distinct waves (Figures 5A,B). Analysis of Croatia_Medieval_o samples, representing the earliest medieval Croatian individuals (7th-ninth century), reveals complex admixture patterns (Figure 5A). The optimal four-way model identifies Latvia_BA (Baltic) contributing 50.8%, Kazakhstan Sarmatian contributing 17.3%, Balkans_IA contributing 19.1%, and Steppe components contributing 12.8%. These high Baltic ancestry levels in early samples contrast with later medieval populations, supporting a multi-wave model with progressive admixture. The temporal flow diagram illustrates component transformations (Figure 5B).

**FIGURE 6:**
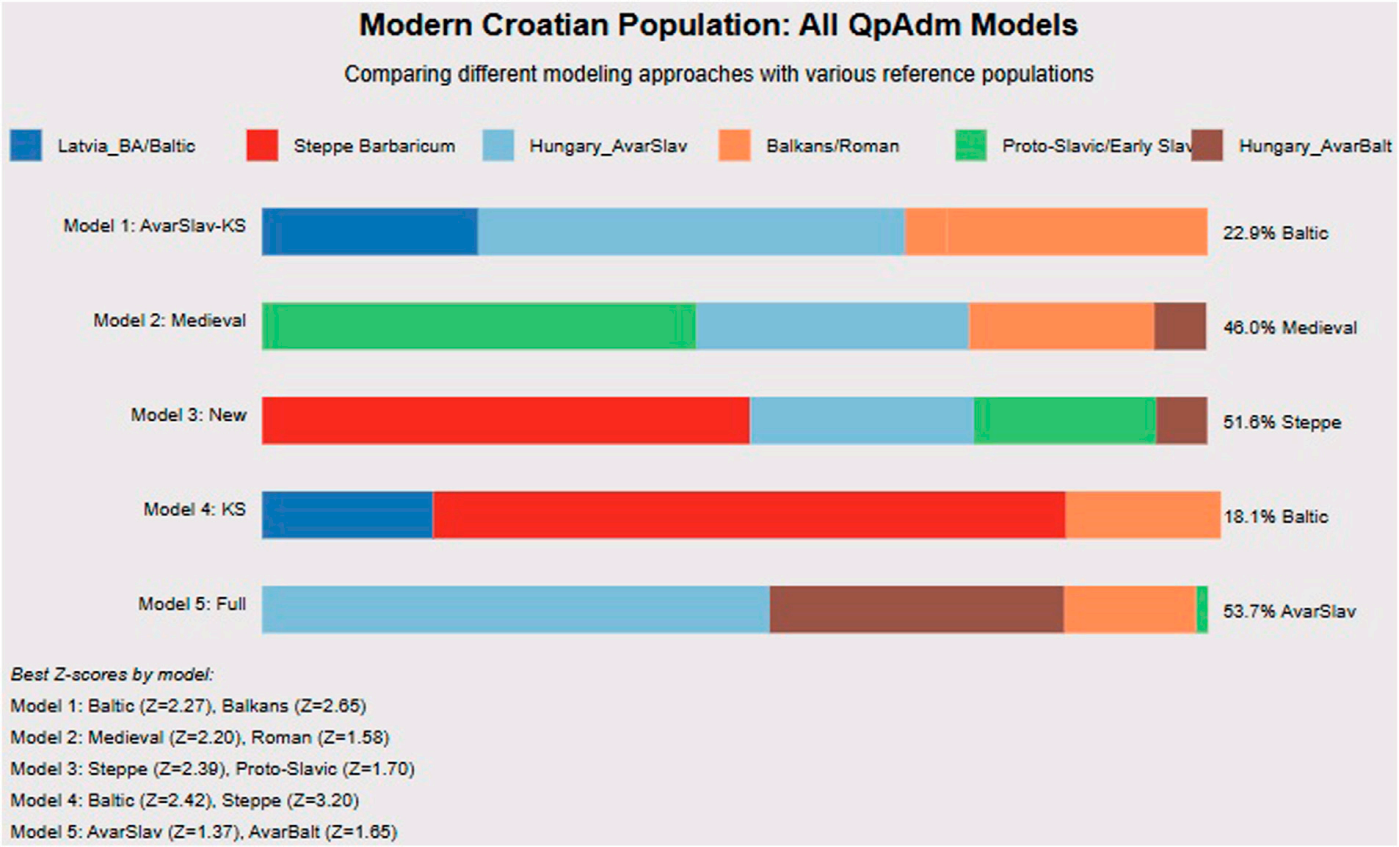
Modern Croatian Population: Comprehensive QpAdm Analysis. In this fugure we present five different admixture models for contemporary Croatian populations, demonstrating the complexity of Croatian genetic heritage. Model 2 (Medieval Model) shows the highest contribution from Croatia_Medieval_o (46%), with additional Roman, Proto-Slavic, and Hungary_AvarBalt components. Model 3 (New Model) emphasizes Steppe Barbaricum ancestry (51.6%), highlighting the Barbaricum’s long-term demographic impact. Model 5 (Full Model) achieves the best statistical fit with 53.7% combined Avar-period ancestry (Hungary_AvarSlav), confirming that interactions within the Avar Khaganate fundamentally shaped Croatian genetic structure. The consistency across models—showing 20%–25% pre-Slavic Balkan ancestry and 50%–60% various Slavic components—supports our multi-wave migration model. Z-scores >2 across multiple models confirm statistical significance. These results demonstrate that modern Croatian populations formed through integration of at least three distinct Slavic waves with local Balkan populations.

Five different admixture models for contemporary Croatian populations converge on consistent patterns (Figure 6). Model 1 (AvarSlav focus) shows 22.9% Baltic, 37.1% Hungary_AvarSlav, and 40% Balkans/Roman ancestry. Model 2 (Medieval focus) reveals 46% Croatia_Medieval_o, 29% Roman, 20% Proto-Slavic, and 5% Baltic components. Model 3 (Steppe focus) identifies 51.6% Steppe Barbaricum, 27% Early Slavic, and 21.4% Balkans ancestry. Model 5 (Full model, best fit) demonstrates 53.7% combined Avar-period ancestry and 46.3% other components.

The consistency across models, showing 20%–25% pre-Slavic Balkan ancestry and 50%–60% various Slavic components, supports multiple migration waves with varying degrees of admixture. The temporal flow diagram illustrates how these components transformed across periods.

The evidence supports at least three distinct migration phases.

1. Early wave (6th century): High Baltic ancestry (>50%), minimal local admixture, documented by Gepid sources mentioning Slavs crossing at Iron Gates
2. Avar-associated wave (6th-7th century): Mixed Avar-Slavic profiles (Supplementary Figure S8), 25%–30% Baltic ancestry, substantial Byzantine admixture
3. Late wave (8th-9th century): Associated with traditional Croatian migration narratives, intermediate Baltic ancestry (35%–40%)

## 5 Discussion

### 5.1 Overview of findings

Our systematic analysis of 1,800 ancient genomes provides unprecedented resolution of the complex population dynamics underlying Croatian ethnogenesis. By developing a methodological framework that overcomes the Germanic-Slavic discrimination problem through systematic principal component screening, we demonstrate that South Slavic genetic structure emerged through multiple waves of migration and extensive population interaction within the Avar Khaganate, rather than through simple replacement or single migration events. The identification of PC9 as a discriminating component represents a methodological advance applicable to other cases where populations share dominant ancestry components but differ in marginal genetic signatures.

The evidence for 50%–60% Slavic ancestry in modern Croatian populations, retained through at least three distinct migration waves between the 6th and 8th centuries CE, fundamentally revises our understanding of South Slavic ethnogenesis. Rather than the single migration event suggested by Constantine VII Porphyrogenitus’s account, the genetic data reveals a complex process of sequential population movements, each contributing distinct genetic and cultural elements to the emerging Croatian identity.

### 5.2 Comparison with global population studies

Our findings align with recent large-scale ancient DNA studies (Sarkissian et al., 2022), while providing novel regional insights. The 50%–60% Slavic ancestry identified in modern Croatians exceeds the 30%–60% range reported by Olalde et al. for general Balkan populations, likely due to our improved detection method using marginal principal components. This level of genetic turnover parallels other major demographic transformations (Török, 2022) in European prehistory.

Similar population replacement levels have been documented in other contexts: the Anglo-Saxon migration to Britain showed 40%–75% continental ancestry (Gretzinger et al., 2022), while the Hungarian Conquerors contributed 30%–40% ancestry to the Carpathian Basin (Maár et al., 2021). Unlike the strongly male-biased migrations documented in Bronze Age Iberia (Olalde et al., 2023) and Anglo-Saxon Britain, our evidence suggests more balanced Slavic migrations with both male and female contributors, consistent with family-group movements rather than military campaigns alone.

The Baltic ancestry gradient we document (71% in Lithuanians decreasing to 39%–51% in early Croatians) provides quantitative support for the linguistic homeland hypothesis, comparable to the steppe ancestry gradients that track Indo-European expansions (Haak et al., 2015; Allentoft et al., 2015). This pattern resembles the dilution of Yamnaya ancestry from 75% in Corded Ware populations to 30%–50% in modern Europeans (Lazaridis et al., 2022), suggesting similar processes of sequential admixture during population expansions.

### 5.3 The steppe barbaricum as demographic crucible

The extraordinary genetic diversity (Supplementary Figure S3) documented in the Steppe Barbaricum between the 1st and fifth centuries CE reveals a previously unrecognized zone of intensive population interaction. The balanced representation of five distinct ancestral components—Germanic, Proto-Slavic, Sarmatian, Byzantine, and Balkan Iron Age—each contributing approximately 20% ancestry, has few parallels in ancient DNA studies.

This level of population integration exceeds even the cosmopolitan profiles documented in Roman frontier cities (Veeramah et al., 2018) or trading centers like Viking Age Birka (Krzewińska et al., 2018). The presence of Proto-Slavic components in fifth-century Barbaricum samples predates traditional historical accounts by over a century, suggesting either earlier migrations than documented or *in situ* development of Slavic identity through ethnogenesis processes similar to those proposed for other barbarian groups (Geary, 1999; Pohl, 2018).

The Barbaricum’s role as a demographic source contributing 20%–40% ancestry to multiple later populations parallels the function of other frontier zones in facilitating ethnic transformations. Similar processes have been documented in the Middle Danube region during the Bronze Age (Gerber et al., 2023) and in the Pontic-Caspian steppe during Scythian formations (Järve et al., 2019).

### 5.4 Reconsidering the Avar Khaganate’s social structure

The identification of individuals with predominantly Slavic genetic profiles in elite Avar burials fundamentally challenges traditional interpretations of rigid ethnic hierarchies within the Khaganate. This finding parallels recent discoveries in other nomadic empires: the Xiongnu confederation included genetically diverse elites (Jeong et al., 2020), while the Mongol Empire incorporated local elites across its territories (Damgaard et al., 2018).

The substantial Byzantine genetic component (15%–20%) in Avar-period Slavic profiles indicates ongoing interaction with imperial territories despite political tensions. This pattern resembles the genetic continuity documented across the Roman-Medieval transition in other frontier regions (Amorim et al., 2018) and suggests that political boundaries did not prevent population movement and intermarriage.

The presence of individuals with predominantly Baltic ancestry (59%–75%) in Avar contexts, exemplified by sample CSB-9. SG, provides crucial evidence for understanding pre-expansion Slavic genetic structure. These individuals may represent specialized military recruits, similar to the Varangians in Byzantine service or the diverse warrior bands documented in Viking Age Scandinavia (Margaryan et al., 2020).

### 5.5 Implications for understanding Croatian ethnogenesis

The multi-wave migration model emerging from genetic data aligns with fragmented historical accounts while revealing previously unknown complexity. The three distinct waves we identify - early (6th century, >50% Baltic ancestry), Avar-associated (6th-7th century, 25%–30% Baltic), and late (7th-8th century, 35%–40% Baltic) suggest that Croatian ethnogenesis involved sequential integration of related but distinct Slavic groups.

This pattern contrasts with single-event models of ethnic formation and instead supports processual models emphasizing gradual transformation. However, unlike purely constructivist approaches that minimize demographic change, our data demonstrates substantial population replacement accompanied by cultural transformation. The persistence of 20%–25% pre-Slavic Balkan ancestry indicates that Croatian formation involved integration rather than complete replacement, similar to patterns documented in Anglo-Saxon England (Gretzinger et al., 2022) and Viking Age Ireland (Margaryan et al., 2020).

The emergence of key South Slavic genetic features within the Avar Khaganate, rather than after Balkan settlement, suggests that interactions in the Carpathian Basin fundamentally shaped South Slavic identity. This finding supports recent archaeological arguments for the Carpathian Basin as a crucible of early medieval ethnic formations (Csaki et al., 2020) and highlights the importance of the Avar period in European demographic history.

### 5.6 Methodological implications for ancient DNA studies

Our systematic screening approach for marginal principal components addresses a fundamental challenge in population genetics: distinguishing closely related populations that share dominant ancestry sources. The success of PC9 in discriminating Slavic from Germanic populations, despite their shared Baltic Bronze Age heritage, demonstrates that informative genetic variation often resides in components capturing minimal overall variance.

This methodology has broad applicability for studying other cases of populations with shared major ancestry but distinct demographic histories.

### 5.7 Historical and archaeological correlations

The genetic evidence provides new context for interpreting historical sources and archaeological patterns. The presence of Slavic genetic signatures in the Barbaricum by the fifth century CE correlates with Jordanes’s mentions of “Sclaveni” north of the Danube (551 CE) and supports earlier presence than traditionally assumed. The identification of Slavs in elite Avar burials aligns with Byzantine sources mentioning Slavic leaders operating within the Avar confederation (Menander Protector, 6th century).

Archaeological patterns of material culture change in the 6th-7th centuries, particularly the spread of Prague-type pottery and sunken-floored dwellings, can now be understood as reflecting substantial demographic change rather than merely cultural diffusion. The genetic evidence for multiple migration waves correlates with the archaeological identification of distinct pottery traditions and burial customs appearing sequentially rather than simultaneously (Djino, 2010).

### 5.8 Limitations and future directions

While our analysis provides unprecedented resolution of Croatian ethnogenesis, several limitations should be acknowledged. The systematic PC screening approach, while effective for this case, requires validation in other population contexts. The specific principal component capturing discriminating ancestry may vary depending on the populations included in analysis, necessitating systematic screening rather than assuming PC9 will universally capture Slavic ancestry.

Temporal resolution remains limited by the availability of well-dated samples from crucial transitional periods, particularly the fifth-6th centuries CE. Future studies incorporating more samples from this period could refine our understanding of the timing and tempo of population changes. Additionally, integrating uniparental markers (Y-chromosome and mitochondrial DNA) could provide insights into sex-biased migration patterns and social organization.

The complex demographic history revealed here suggests that other European populations traditionally viewed as resulting from simple migration events may similarly show evidence of multiple waves and extensive admixture when analyzed with appropriate methods. Future studies should apply similar systematic approaches to investigate ethnogenesis processes in other regions where historical and archaeological evidence suggests complex population formations.

## 6 Conclusion

This study demonstrates that Croatian ethnogenesis resulted from complex demographic processes involving at least three distinct waves of Slavic migration between the 6th and 8th centuries CE, each contributing unique genetic signatures to the emerging population. Through systematic screening of marginal principal components, we overcome longstanding methodological challenges in distinguishing Slavic from Germanic ancestry, revealing that 50%–60% of modern Croatian ancestry derives from Slavic sources while 20%–25% represents pre-Slavic Balkan continuity.

The identification of Proto-Slavic components in fifth-century Steppe Barbaricum populations and Slavic individuals in elite Avar burials revises traditional narratives of South Slavic origins. Our findings indicate that key aspects of South Slavic genetic structure emerged through population interactions within the Avar Khaganate rather than post Balkan settlement, highlighting the Carpathian Basin’s role as a crucial demographic interface in early medieval Europe.

The methodological framework developed here combines systematic PC screening with formal admixture modeling and suggests a template for resolving similar challenges in ancient DNA studies where populations share dominant ancestry components. These approaches enable detection of cryptic population relationships crucial for understanding complex ethnogenesis processes.

## Data Availability

Data is publicly available at: https://dataverse.harvard.edu/dataset.xhtml?persistentId=doi:10.7910/DVN/FFIDCW.

## Supplementary material

SUPPLEMENTARY TABLE S1 PC9 Extremes Analysis. This table presents the systematic screening results of Principal Component 9 (PC9) values across 1,800 ancient DNA samples, identifying populations with the highest PC9 values within European contexts. The analysis reveals that while PC9 reaches maximum values globally in northern Eurasian populations (Nganasan, top row), within European samples it consistently maximizes in early Slavic populations. CEE_EarlyMedieval (Early Western Slavic from Slovakia/Slovenia, 6th-9th century CE) shows the highest European values (0.0186), followed by various Croatian medieval samples (0.0162–0.0158). The presence of Latvia_BA samples with high PC9 values confirms the Baltic Bronze Age connection to early Slavic populations. This finding is methodologically significant as it demonstrates that PC9, typically a marginal component capturing <1% of total genetic variance, can effectively discriminate Slavic from Germanic populations despite their shared Baltic Bronze Age ancestry—a discrimination impossible using conventional PC1-2 analysis. The cryptic Slavic signal search results (bottom panel) confirm successful identification of previously unrecognized Slavic individuals in various archaeological contexts, validating our systematic PC screening approach.

SUPPLEMENTARY FIGURE S1 Standard PC1-2 Analysis. Conventional Principal Component Analysis (PC1 vs. PC2) demonstrating the Germanic-Slavic discrimination problem. Despite capturing the greatest genetic variance, PC1-2 fails to separate Germanic from Slavic populations due to shared Baltic Bronze Age ancestry, necessitating our systematic screening approach to identify discriminating marginal components.

SUPPLEMENTARY FIGURE S2 Genetic Distance Heat Maps. The heat maps visualize Euclidean genetic distances between ancient and modern Slavic populations using two different PC combinations: PC9,4,3 space (left) and PC1,2,3 space (right). Red indicates closer genetic affinity while blue indicates greater distance. In PC9,4,3 space, which optimizes for Slavic ancestry detection, clear clustering patterns emerge: Latvia_BA shows minimal distance to itself (red diagonal), while Hungarian Avar-period Slavic samples (Hungary_EarlyAvarslav, Hungary_LateAvarslav) cluster tightly with Croatian medieval samples. The CEE_EarlyMedieval population shows intermediate distances to most groups, consistent with its role as a source population. Importantly, modern populations (Croatian.HO, Bulgarian.HO) maintain strong connections to medieval predecessors. The contrast with PC1,2,3 space demonstrates why marginal PC analysis is essential—conventional PC space fails to resolve these fine-scale population relationships. This methodological validation supports our systematic approach to detecting cryptic ancestry patterns in ancient DNA datasets.

SUPPLEMENTARY FIGURE S3 Steppe Barbaricum Population Components (fifth century CE). This pie chart and bar graph present qpAdm modeling results for 30 individuals from the Steppe Barbaricum region (Hungarian Plain between Danube and Tisza rivers, 1st-5th century CE). The remarkably balanced distribution of five ancestral components—Germanic (21.5%), Balkan Iron Age (20.8%), Proto-Slavic (20.2%), Byzantine (19.1%), and Steppe Sarmatian-Hunnic (16.9%)—reveals an extraordinarily integrated multicultural society. This finding is crucial for understanding Croatian ethnogenesis as it demonstrates that the Barbaricum functioned as a demographic crucible where population mixing occurred centuries before the traditional dating of Slavic migrations. The presence of substantial Proto-Slavic ancestry (20.2%) in 5th-century samples predates historical accounts of Slavic presence by over a century, suggesting either earlier migrations than documented or *in situ* development of Slavic genetic signatures. This admixed population later contributed 20%–40% ancestry to Early Western Slavic groups and approximately 20% to medieval Croatian populations, indicating the Barbaricum’s pivotal role in shaping South Slavic genetic structure.

SUPPLEMENTARY FIGURE S4 Evolution of Population Components Over Time. This stacked bar chart traces the transformation of population components across three critical periods in the Steppe Barbaricum region. The Early Period (Chernyakhiv/Scythian dominance) shows predominantly steppe ancestry (>60%) with minor Scythian, Byzantine, and Proto-Slavic elements. The Mixed Period (Germanic/Sarmatian interaction) demonstrates dramatic demographic transformation with Sarmatian components comprising ∼35%, Germanic ∼20%, and emerging Avar-Slavic signatures (∼15%). The Later Period (Croatian Formation) reveals the establishment of the medieval genetic structure with Steppe Barbaricum contributing ∼50%, significant Avar-Slavic presence (∼25%), Baltic-like ancestry (∼10%), and persistent Proto-Slavic elements (∼15%). Model fit p-values (shown but not specified in detail) validate these admixture models. This temporal progression directly supports the multi-wave migration model for Croatian ethnogenesis, demonstrating that population formation occurred through gradual integration rather than sudden replacement, with each period contributing distinct genetic layers to the final medieval Croatian genetic profile.

SUPPLEMENTARY FIGURE S5 MDS Plot. This scatter plot displays the first two dimensions (V1 and V2) from multidimensional scaling analysis of genetic distances between key populations. Contemporary populations (red dots) including Croatian.HO, Polish.DG, Czech.HO, and Bulgarian.HO cluster in the upper right quadrant. Reference populations (cyan dots) show clear separation: Latvia_BA occupies an extreme position on the left, confirming its distinctive Baltic Bronze Age signature, while CEE_EarlyMedieval and Croatia_Medieval_o occupy intermediate positions between Baltic and contemporary populations. The Hungarian Avar-period samples (Hungary_EarlyAvarslav, Hungary_LateAvarslav, Hungary_Avar_5) form a distinct cluster, demonstrating their unique admixed profile. Hungary_EarlyAvarBalt’s position near Latvia_BA confirms the presence of individuals with predominantly Baltic ancestry within the Avar Khaganate (Supplementary Figure S8). This spatial arrangement reveals three major genetic poles: Baltic (ancestral), Avar-Slavic (intermediate), and modern Slavic (derived), supporting our model of gradual admixture and population transformation rather than simple replacement.

SUPPLEMENTARY FIGURE S6 Distance Analyses Using Different PCs. Alternative distance analyses using different PC combinations. PC1,2,3 space showing limited population discrimination. Diagonal deviation analysis. Additional validation of PC9-based discrimination. These analyses confirm the robustness of our marginal PC approach across multiple analytical frameworks.

SUPPLEMENTARY FIGURE S7 Hungary_AvarBalt Individual Models. This figure presents two alternative admixture models for a specific individual (Hungary_AvarBalt) from the Avar Khaganate, demonstrating the robustness of Baltic ancestry detection. The 4-way model identifies 59.1% Latvia_BA ancestry alongside minor contributions from Steppe Barbaricum, Kazakhstan Sarmatian, and Balkans IA populations. The simpler 2-way model suggests even higher Baltic ancestry (73.8%). Standard errors and Z-scores confirm statistical significance (Z > 3 indicates strong support). This individual, found in an Avar-period archaeological context, represents either direct Baltic migration to the Khaganate or preservation of unmixed early Slavic populations. The finding challenges traditional narratives of strict ethnic hierarchies within the Avar Khaganate and supports our discovery of Slavic individuals in elite burial contexts. The high Baltic component in this individual provides a genetic “benchmark” for understanding pre-admixture Slavic genetic structure.

SUPPLEMENTARY FIGURE S8 Avar-Slavic Population Components. This pie chart and bar graph present the genetic composition of Avar-period Slavic populations based on qpAdm analysis with statistical validation. Early Western Slavic ancestry dominates (59.2%), confirming the primarily Slavic character of these individuals despite their Avar archaeological context. Roman-Byzantine contribution (20.9%) indicates substantial interaction with imperial populations, while Steppe Post-Hunnic ancestry (19.9%) reflects integration with steppe peoples. The absence of Steppe Barbaricum ancestry (0.0%) is notable. Z-scores >3 for Early Western Slavic ancestry confirm strong statistical support for this being the primary component. This composition demonstrates that Slavic populations within the Avar Khaganate maintained their distinct genetic identity while incorporating Byzantine and steppe elements, creating the specific Avar-Slavic genetic profile that would contribute approximately 29% ancestry to medieval Croatian populations.

## References

[B1] Allentoft M. E. Sikora M. Sjögren K. G. Rasmussen S. Rasmussen M. Stenderup J. (2015). Population genomics of Bronze Age eurasia. Nature N 522 (7555), 167–172. 10.1038/nature14507 26062507

[B2] Amorim C. Vai S. Posth C. Modi A. Koncz I. Hakenbeck S. (2018). Understanding 6th-century barbarian social organization and migration through paleogenomics. Nat. Commun. N 9 (1), 3547. 10.1038/s41467-018-06024-4 30206220 PMC6134036

[B3] Cavalli-Sforza L. L. (1994). History and geography of human genes. Princeton: Princeton University Press.

[B4] Chobanov T. (2021). The debate about the origin of the protobulgarians in the beginning of 21st c. Sofia.

[B5] Constantine Porphyrogenitus. De Administrando Imperio (1967). Dumbarton oaks texts CFHB, 1 Washington, USA

[B6] Csáky V. Gerber D. Koncz I. Csiky G. Mende B. G. Szeifert B. (2020). Genetic insights into the social organisation of the Avar period elite in the 7th century AD carpathian basin. Sci. Rep. N 10 (1), 948. 10.1038/s41598-019-57378-8 31969576 PMC6976699

[B7] Damgaard P. Marchi N. Rasmussen S. Peyrot M. Renaud G. Korneliussen T. (2018). The first horse herders and the impact of early Bronze Age steppe expansions into Asia. Science N 360, eaar7711. 10.1126/science.aar7711 29743352 PMC6748862

[B8] Djino D. (2010). Becoming slav, becoming croat. Identity transformations in post-roman and early medieval dalmatia. Leiden-Boston: Brill.

[B9] Freilich S. Ringbauer H. Los D. Novak M. Pavičić D. T. Schiffels S. (2021). Reconstructing genetic histories and social organisation in Neolithic and Bronze Age Croatia. Sci. Rep. N 11 (1), 16729. 10.1038/s41598-021-94932-9 34408163 PMC8373892

[B10] Geary P. (1999). “Barbarians and ethnicity,” in Late antiquity. Editors Brown P. Bowersock G. Grabar A. (Cambridge MA), 106–129.

[B11] Gerber D. Szeifert B. Székely O. Egyed B. Gyuris B. Giblin J. I. (2023). Interdisciplinary analyses of Bronze Age communities from Western Hungary reveal complex population histories. Mol. Biol. Evol. 40 (9), 182. 10.1093/molbev/msad182 37562011 PMC10473862

[B12] Gnecchi-Ruscone G. Khussainova E. Kahbatkyzy N. Musralina L. Spyrou M. A. Bianco R. A. (2021). Ancient genomic time transect from the Central Asian steppe unravels the history of the scythians. Sci. Adv. 7, 4414. 10.1126/sciadv.abe4414 33771866 PMC7997506

[B13] Gnecchi-Ruscone G. Szécsényi-Nagy A. Koncz I. Csiky G. Rácz Z. Rohrlach A. B. (2022). Ancient genomes reveal origin and rapid Trans-Eurasian migration of 7th century Avar elites. Cell N 185, 1402–1413.e21. 10.1016/j.cell.2022.03.007 35366416 PMC9042794

[B14] Gretzinger J. Sayer D. Justeau P. Altena E. Pala M. Dulias K. (2022). The Anglo-Saxon migration and the formation of the early English gene pool. Nature N 610 (7930), 112–119. 10.1038/s41586-022-05247-2 36131019 PMC9534755

[B15] Haak W. Lazaridis I. Patterson N. Rohland N. Mallick S. Llamas B. (2015). Massive migration from the steppe was a source for Indo-European languages in Europe. Nature N 522 (7555), 207–211. 10.1038/nature14317 25731166 PMC5048219

[B16] Harney E. Patterson N. Reich D. Wakeley J. (2021). Assessing the performance of qpAdm: a statistical tool for studying population admixture. Genetics N 217 (4), iyaa045. 10.1093/genetics/iyaa045 33772284 PMC8049561

[B17] Istvánovits E. (1998). Some considerations about the religion, tribal affiliation and chronology of the sarmatians of the great Hungarian plain. Annali N 58, 193–228.

[B18] Istvánovits E. (2020). Sarmatians on the borders of the roman empire: steppe traditions and imported cultural phenomena. Anc. Civilizations Scythia Sib. N 26, 391–402.

[B19] Järve M. Saag L. Scheib C. L. Pathak A. K. Montinaro F. Pagani L. (2019). Shifts in the genetic landscape of the Western Eurasian steppe associated with the beginning and end of the scythian dominance. Curr. Biol. N 29 (14), 2430–2441.e10. 10.1016/j.cub.2019.06.019 31303491

[B20] Jeong C. Wang K. Wilkin S. Taylor W. T. T. Miller B. K. Bemmann J. H. (2020). A dynamic 6,000-Year genetic history of eurasia's eastern steppe. Cell N 183 (4), 890–904. 10.1016/j.cell.2020.10.015 33157037 PMC7664836

[B21] Krzewińska M. Kılınç G. M. Juras A. Koptekin D. Chyleński M. Nikitin A. G. (2018). Ancient genomes suggest the eastern Pontic-Caspian steppe as the source of Western Iron Age nomads. Sci. Adv. N 4 (10), eaat4457. 10.1126/sciadv.aat4457 30417088 PMC6223350

[B22] Kulcsár V. Istvánovits E. (2020). New results in the research on the hun age in the great Hungarian plain: some notes on the social stratification of barbarian society. Stud. Uralo-Altaica N 53, 167–182.

[B23] Lazaridis I. Alpaslan-Roodenberg S. Acar A. Açıkkol A. Agelarakis A. Aghikyan L. (2022). The genetic history of the southern arc: a bridge between west Asia and Europe. Science N 377, eabm4247. 10.1126/science.abm4247 36007055 PMC10064553

[B24] Lazaridis I. Alpaslan-Roodenberg S. Acar A. Açıkkol A. Agelarakis A. Aghikyan L. (2022). A genetic probe into the ancient and medieval history of southern Europe and west Asia. Science 377, 940–951. 10.1126/science.abq0755 36007020 PMC10019558

[B25] Lehti S. Vasilyev S. V. Varul L. Kosorukova N. V. Gerasimov D. V. Oshibkina S. V. (2021). Genetic ancestry changes in stone to Bronze Age transition in the east european Plain. Sci. Adv. 7, 6535. 10.1126/sciadv.abd6535 PMC7817100 33523926

[B26] Maár K. Varga G. I. B. Kovács B. Schütz O. Maróti Z. Kalmár T. (2021). Maternal lineages from 10–11th century commoner cemeteries of the carpathian basin. Genes N 12 (3), 460. 10.3390/genes12030460 33807111 PMC8005002

[B27] Maenchen-Helfen O. J. (1973). The world of the huns. University of California Press.

[B28] Maier R. Reich D. (2023). On the limits of fitting complex models of population history to f-statistics. eLife N 12, 1. 10.7554/eLife.85492 PMC10310323 37057893

[B29] Margaryan A. Lawson D. J. Sikora M. Racimo F. Rasmussen S. Moltke I. (2020). Population genomics of the viking world. Nature N 585 (7825), 390–396. 10.1038/s41586-020-2688-8 32939067

[B30] Mathieson I. Alpaslan-Roodenberg S. Posth C. Szécsényi-Nagy A. Rohland N. Mallick S. (2018). The genomic history of southeastern Europe. Nature N 555, 197–203. 10.1038/nature25778 29466330 PMC6091220

[B31] McColl H. Kroonen G. Víctor Moreno-Mayar J. Seersholm F. V. Scorrano G. Pinotti T. (2024). Steppe ancestry in Western eurasia and the spread of the Germanic languages. bioRxiv Cold Spring Harb. Lab. 10.1101/2024.03.13.584607

[B32] Mócsy A. (1974). Pannonia and upper moesia: a history of the middle danube provinces of the roman empire. London and Boston: Routledge and Kegan Paul.

[B33] Novak M. Olalde I. Ringbauer H. Rohland N. Ahern J. Balen J. (2021). Genome-wide analysis of nearly all the victims of a 6200 year old massacre. PLoS One N 16 (3), e0247332. 10.1371/journal.pone.0247332 33690651 PMC7946188

[B34] Olalde I. Carrión P. Mikić I. Rohland N. Mallick S. Lazaridis I. (2023). A genetic history of the balkans from Roman frontier to Slavic migrations. Cell N 186 (25), 5472–5485.e9. 10.1016/j.cell.2023.10.018 38065079 PMC10752003

[B35] Patterson N. Moorjani P. Luo Y. Mallick S. Rohland N. Zhan Y. (2012). Ancient admixture in human history. Genetics N 192-3, 1065–1093. 10.1534/genetics.112.145037 22960212 PMC3522152

[B36] Pohl W. (2018). The avars. A steppe empire in central Europe. Ithaca and London: Cornell University press.

[B37] Sarkissian D. Balanovsky O. Brandt G. Khartanovich V. Buzhilova A. Koshel S. (2022). Ancient DNA reveals prehistoric gene-flow from siberia in the complex human population history of north East Europe. PLoS Genet. N 9, 1. 10.1371/journal.pgen.1003296 PMC3573127 23459685

[B38] Török T. (2022). The genetic origin of huns, avars, and conquering hungarians. Curr. Biol. N 32/13, 2858–2870. 10.1016/j.cub.2022.04.093 35617951

[B39] Veeramah K. Rott A. Groß M. van Dorp L. López S. Kirsanow K. (2018). Population genomic analysis of elongated skulls reveals extensive female-biased immigration in early medieval Bavaria. Proc. Natl. Acad. Sci. N 115 (13), 3494–3499. 10.1073/pnas.1719880115 29531040 PMC5879695

[B40] Vernadsky G. (1959). The origins of Russia. Oxford: Clarendon Press.

