# Supplementary figures and images for: Slavs in the closet: computational genomic analysis reveals cryptic slavic signatures in the Avar Khaganate and their contribution to medieval Croatian population formation
Source: Front Genet. 2025 Sep 22;16:1610942. doi: 10.3389/fgene.2025.1610942 (PMC12498020; doi:10.3389/fgene.2025.1610942)

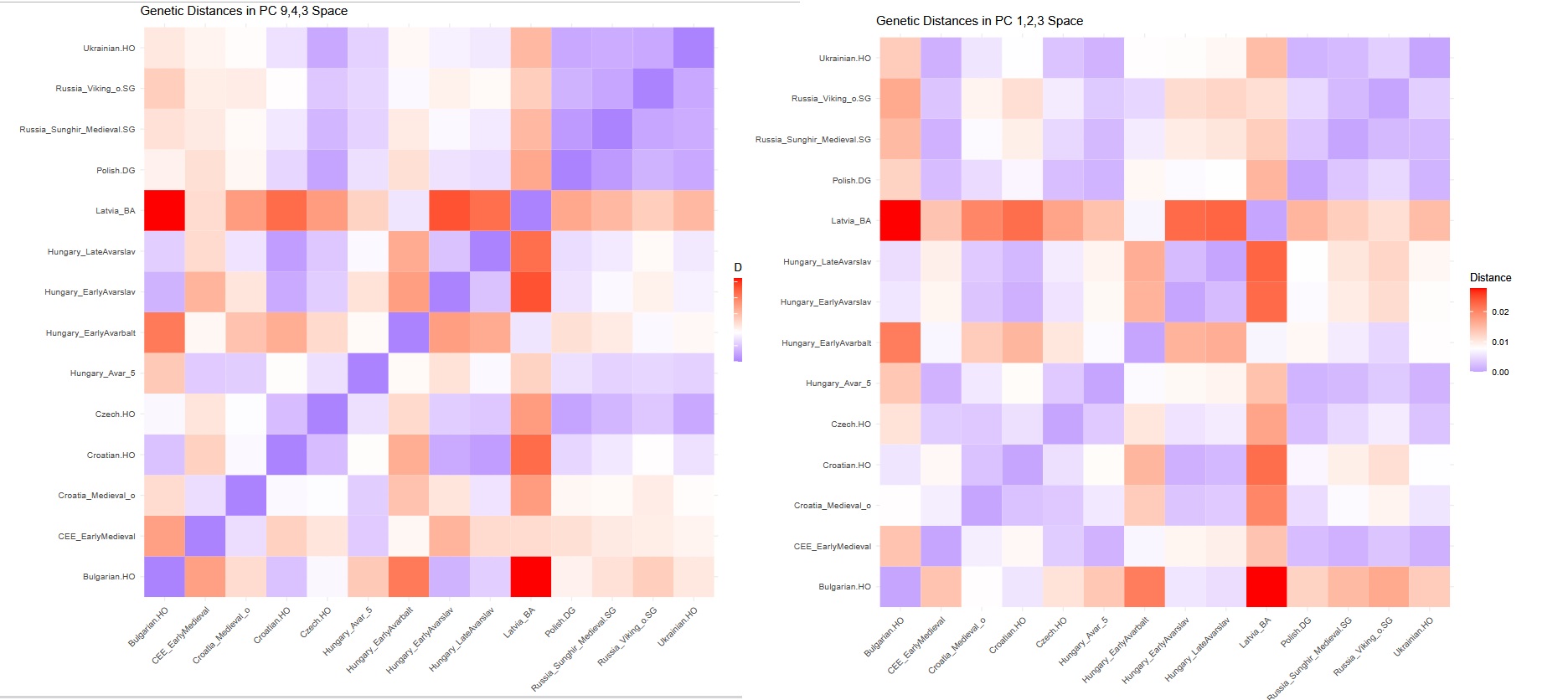

Supplement: Supplementary file 2 [file Image3.jpeg]

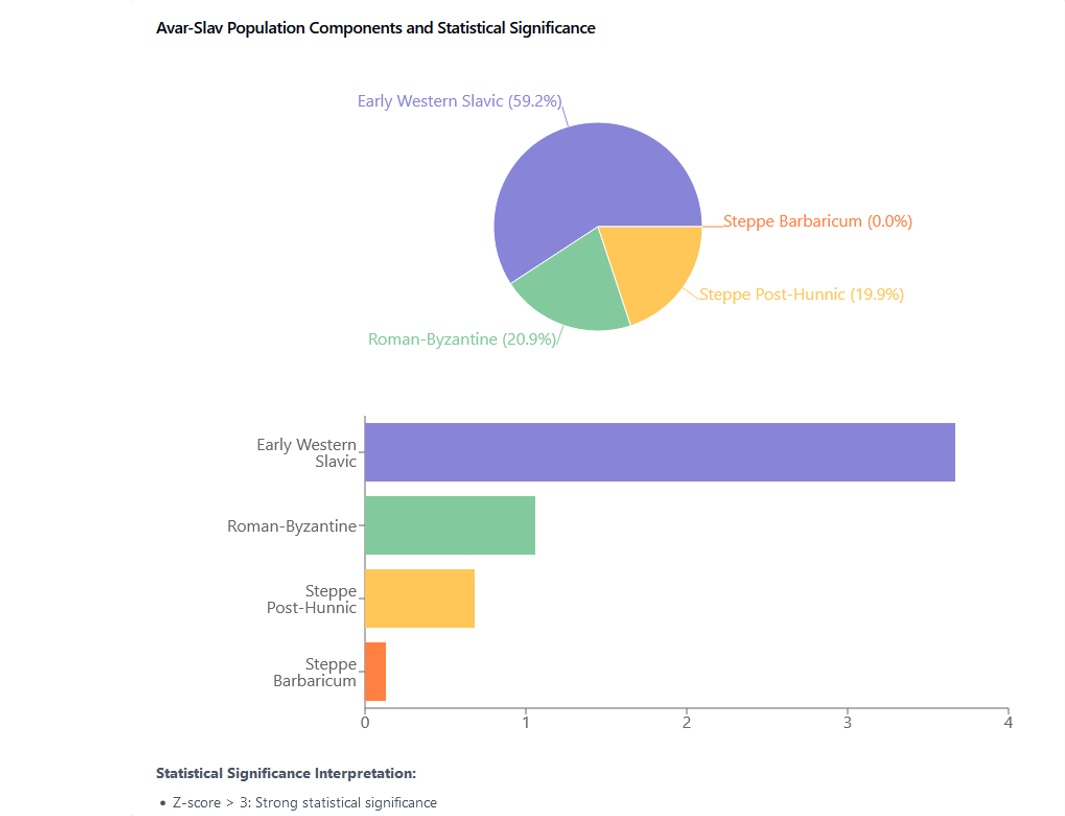

Supplement: Supplementary file 4 [file Image9.jpeg]

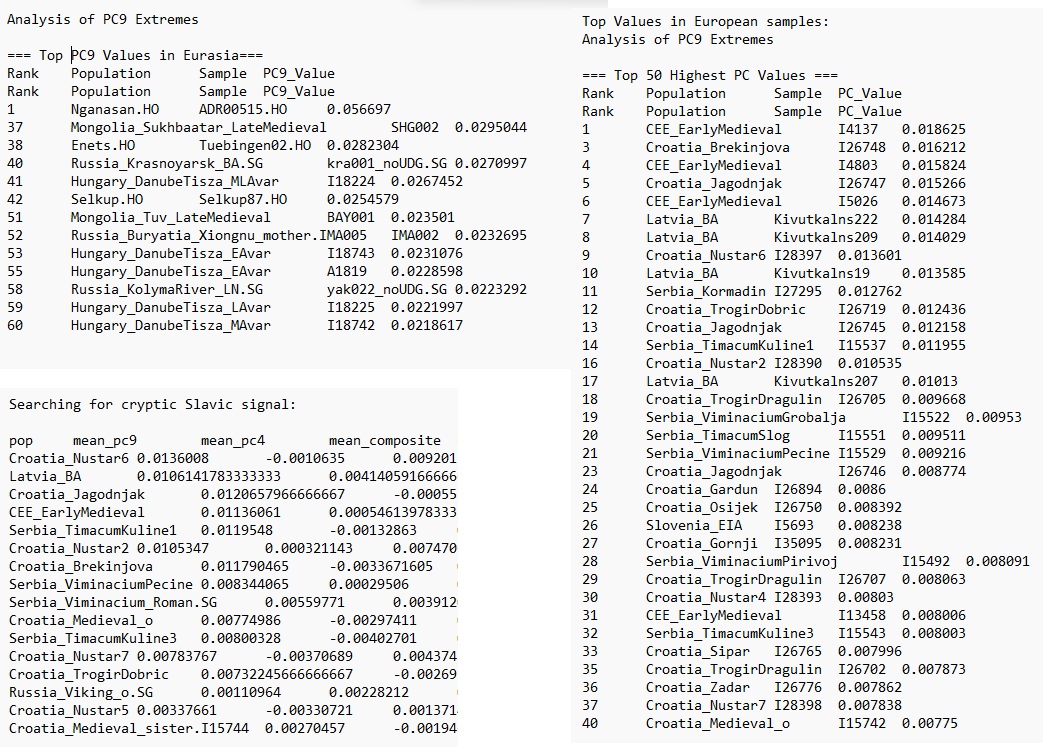

Supplement: Supplementary file 5 [file Image1.jpeg]

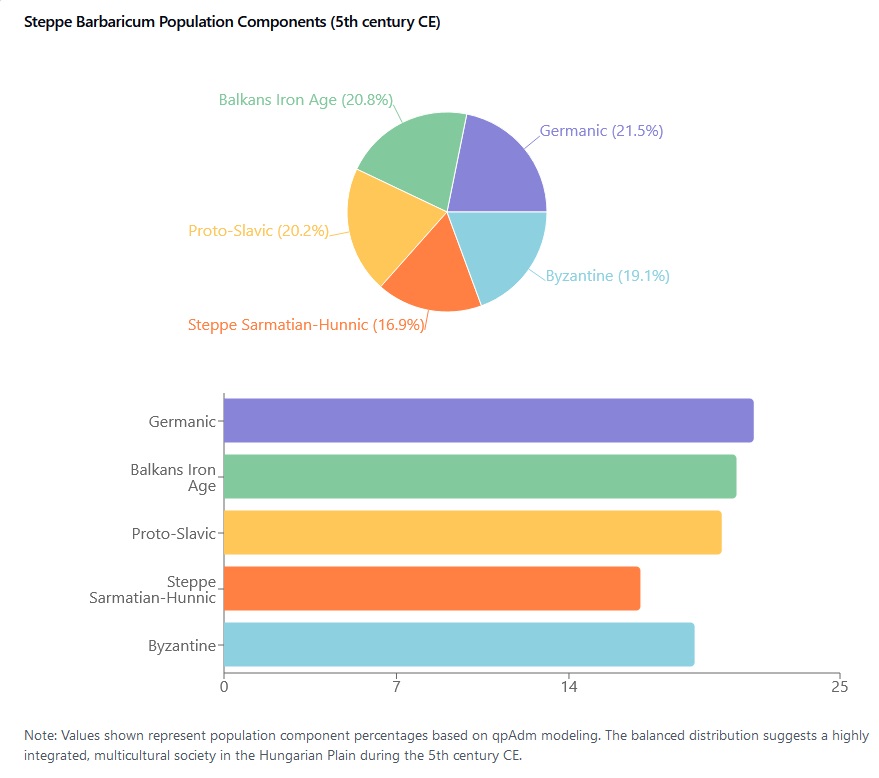

Supplement: Supplementary file 6 [file Image4.jpeg]

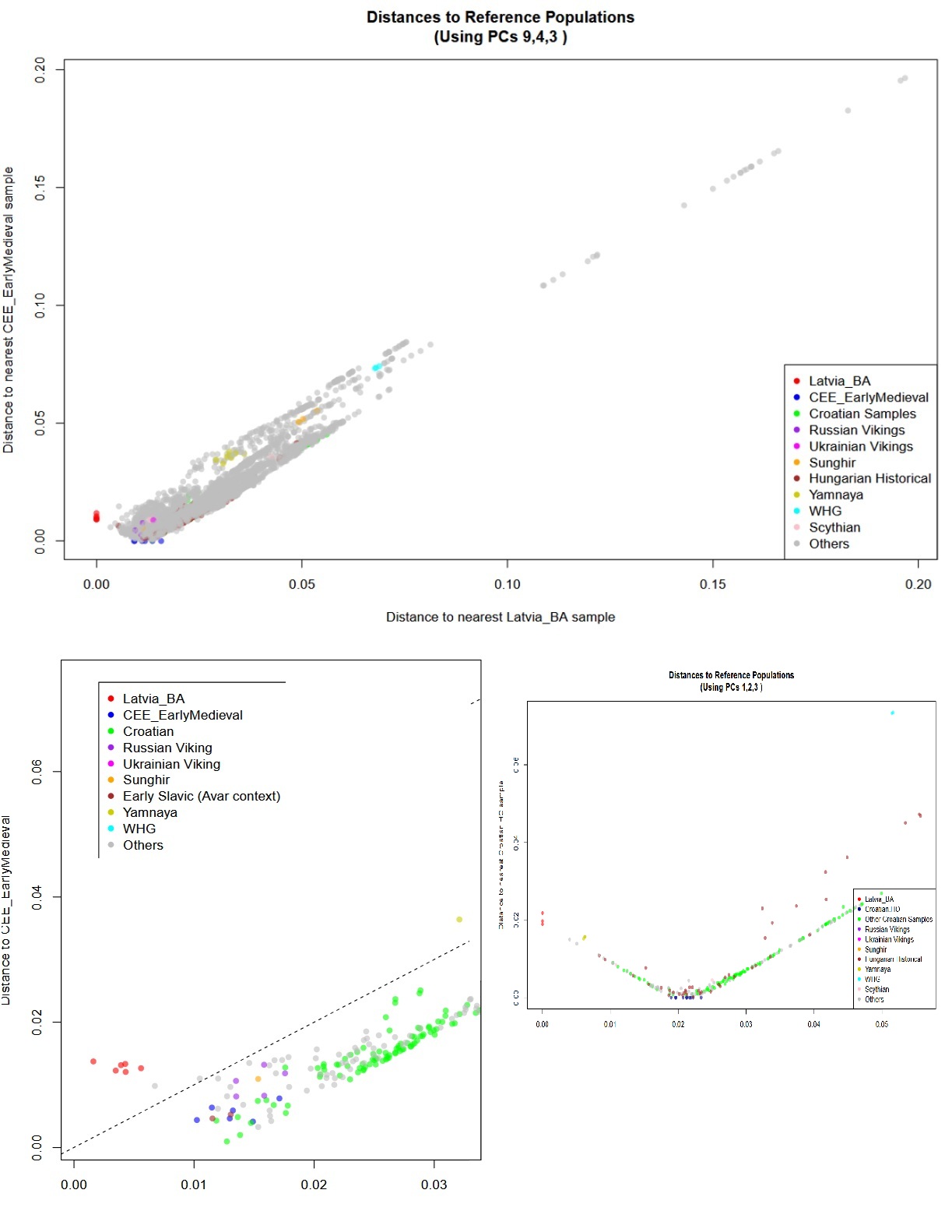

Supplement: Supplementary file 7 [file Image7.jpeg]

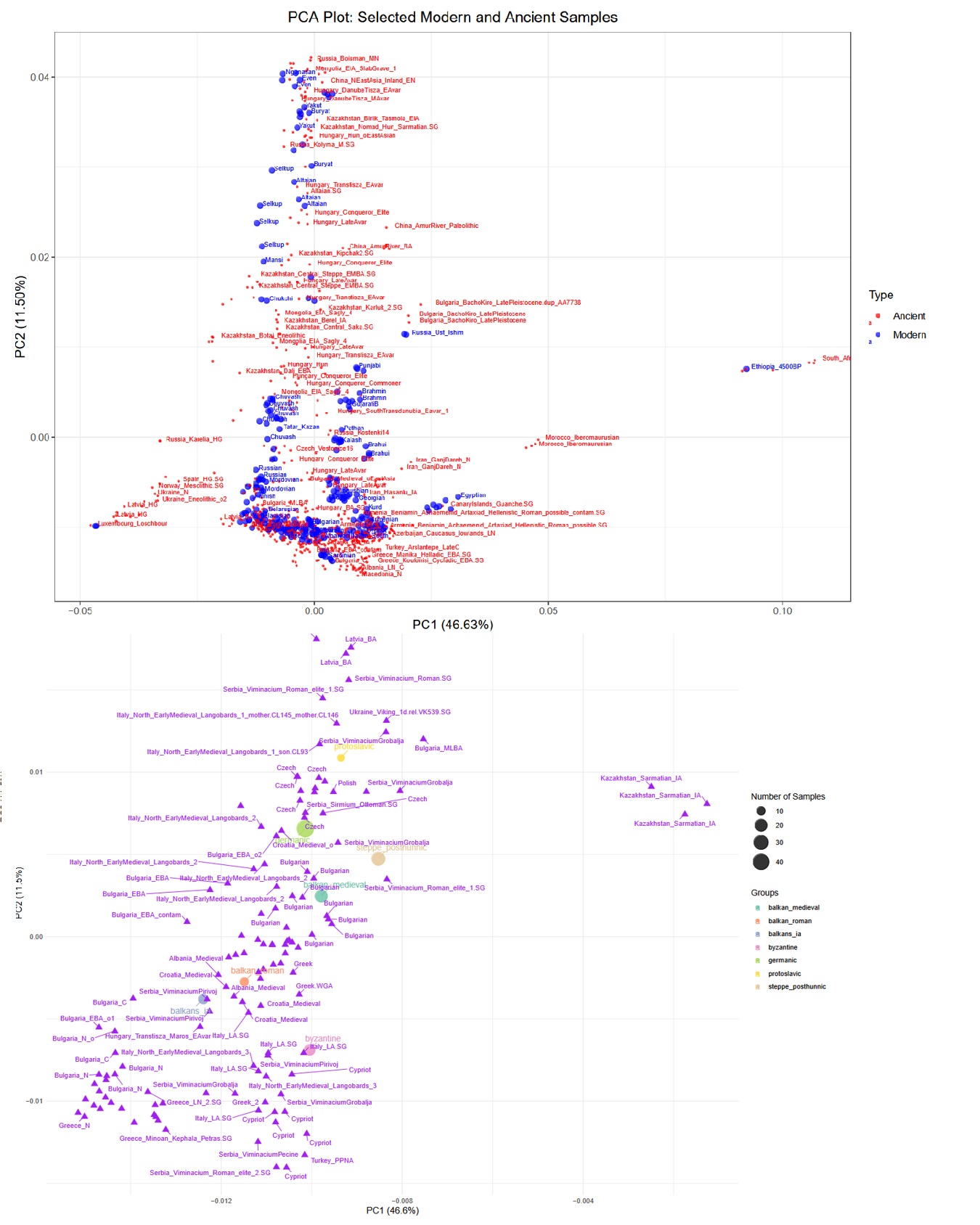

Supplement: Supplementary file 8 [file Image2.jpeg]

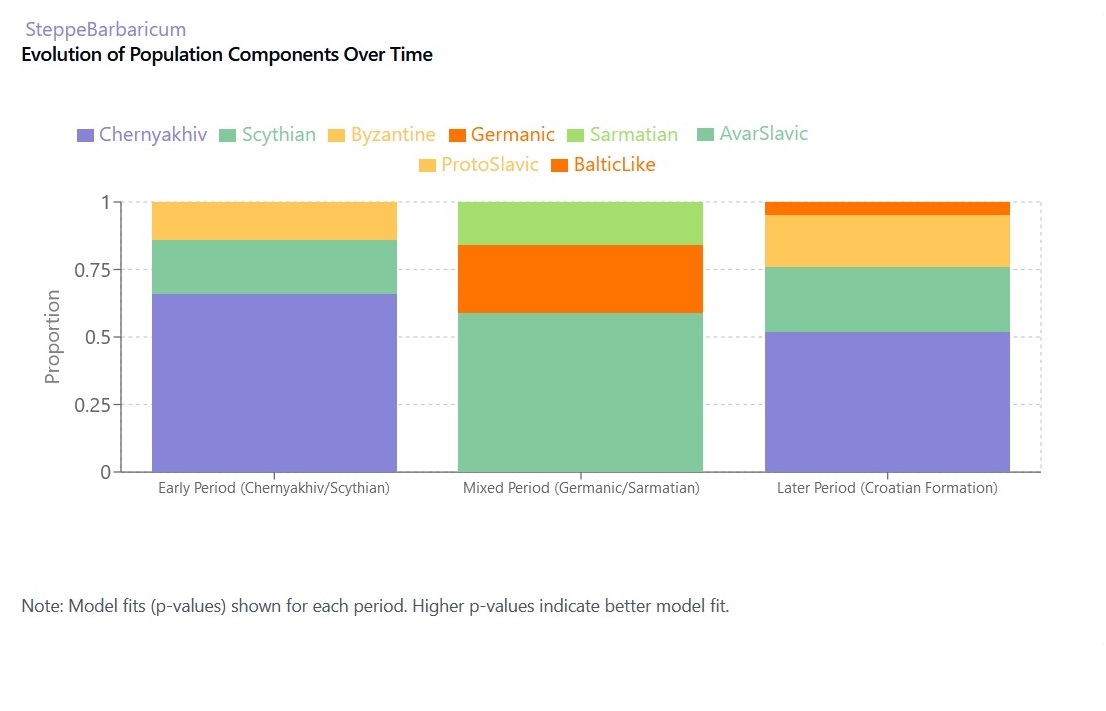

Supplement: Supplementary file 9 [file Image5.jpeg]

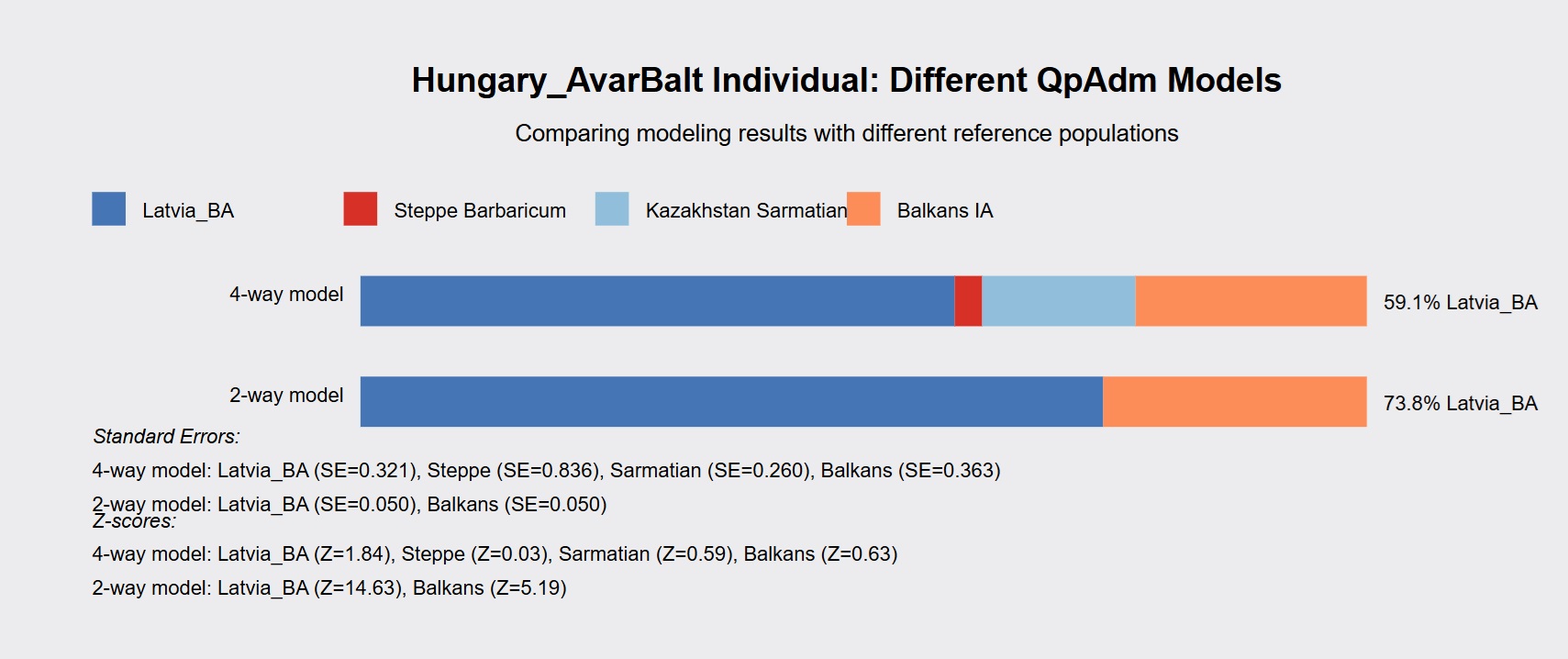

Supplement: Supplementary file 10 [file Image8.jpeg]

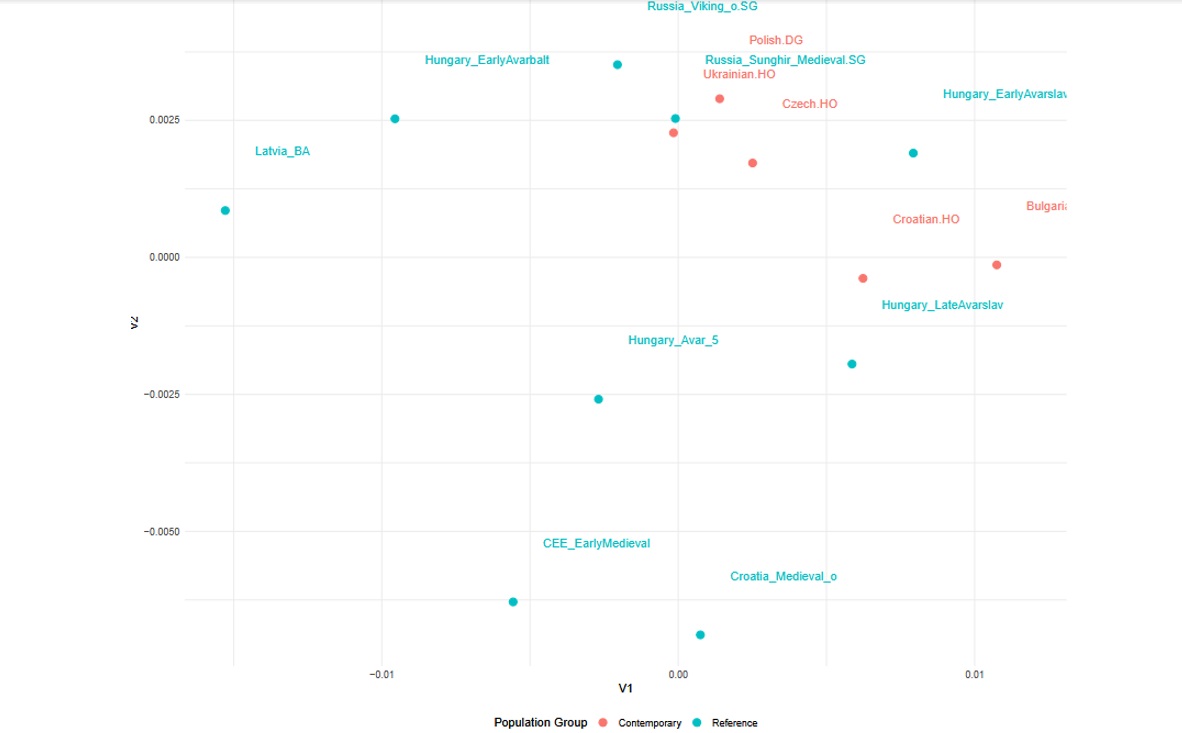

Supplement: Supplementary file 11 [file Image6.jpeg]
