# Supplementary material for: Slavs in the closet: computational genomic analysis reveals cryptic slavic signatures in the Avar Khaganate and their contribution to medieval Croatian population formation
Source: Front Genet. 2025 Sep 22;16:1610942. doi: 10.3389/fgene.2025.1610942 (PMC12498020; doi:10.3389/fgene.2025.1610942)
Supplement: Supplementary file 3 [file Table1.docx]

Additional Information:

Key Individual Samples:

Hungary_AvarBalt (CSB-9.SG):

Adult from Csongrád-Berzsenyi utca

Early Avar period (500-700 CE)

Grave goods: iron buckle, spherical pendant earrings, grey ceramic vessel fragment

E-W oriented grave

Important: Found in a cemetery with earlier Sarmatian graves showing continuity of occupation

Hungary_AvarSlav (SZOD1-829.SG):

Szegvár-Oromdűlő cemetery

Adult (genetically male)

2nd quarter of 7th century CE

Grave goods: knife, earring, spindle-whorl, tube, earpick, cattle bones

Niche-type grave construction

Hungary_AvarSlav (TMH-199.SG):

From Tiszafüred-Majoros-halom

Adult male showing mixed Europid-Mongoloid features

Late Avar period (dated 663-818 calCE)

Grave goods: cast bronze belt fittings, bronze chain, knife, two iron buckles

Hungary_AvarSlav (OBT-56.SG):

From Orosháza

Young adult male with Europid characteristics (Brachycran with Pamirid features)

8th century CE

Grave goods: wood remains with bronze nails, knife, egg, cast bronze belt set with griffin ornaments

Croatia_Medieval_o

7^th^ century CE

Location: Trogir (Split; Byzantine territory)

ID: I15742/2653, grave 6

Female adolescent (16-18 years)

Notable pathologies: developmental malformations in pelvis and femora, trauma evidence

Multiple healed injuries suggesting repeated traumatic events

Key Reference Population Groups:

Balkan Medieval (balkan_medieval):

Bulgaria: Medieval samples from Ryahovo (11th c.), Veliko Tarnovo (12th c.), Samovodene (9th-10th c.)

North Macedonia: Bitola (10th c.)

Serbia: Viminacium-Svetinja (13th c.) - graves with Bulgarian coin imitations

Serbia: Timacum-Kuline (7th c.) - garrison cemetery, evidence of horseback riding

Croatia: Nustar (8th c.) - border fortification between First Bulgarian Empire and Avar Khaganate

Code groups: Bulgaria_Medieval, Bulgaria_Medieval_oEastAsia, Serbia_ViminaciumSvetinja, Serbia_TimacumKuline, Croatia_Nustar, Macedonia_Medieval

Roman Balkans (balkan_roman):

Greece: Marathon area (3rd-4th c.)

Serbia: Viminacium and Rit settlements (2nd-4th c.)

Serbia: Naissus/Niš (4th c.)

Serbia: Sirmium (3rd-4th c.)

Montenegro: Doclea (2nd-4th c.)

Code groups: Greece_Roman, Serbia_ViminaciumRit, Serbia_Naissus_LateAntiquity, Serbia_Sirmium_Roman, Montenegro_Doclea_Roman

Iron Age Balkans (balkans_ia):

Albania: Early Iron Age samples

Bulgaria: Iron Age and Early Iron Age - Kapitan Andreevo, Svilengrad, Eastern Rhodopes (Diamandievo and Stambolovo), Kazanlak area (Rozino, V. Tarnovo)

Macedonia: Iron Age samples

Greece: Mycenaean (12th-16th c. BCE)

Code groups: Albania_BA_IA1, Bulgaria_EIA, Bulgaria_IA, Greece_BA_Mycenaean

Byzantine (byzantine):

Western Anatolia: Nicaea (3rd-5th c.) - 20 samples

Marmara Sea region and Constantinople (4th-7th c.) - 20 samples

Code groups: Turkey_EarlyByzantine, Turkey_Roman

Slavic Groups:

Western Slavic (CEE_EarlyMedieval)

Eastern Slavic (Russia_Sunghir_Medieval)

Steppe Barbaricum (steppe_Barbaricum):

Hungary: Danube-Tisza Late Sarmatian/Early Hun period

Hungary: Hun period samples

Hungary: Trans-Tisza Late Sarmatian/Early Hun

Hungary: Trans-Tisza Roman-Sarmatian period

Ukraine: Western Scythian Iron Age

Ukraine: Scythian samples

Code groups: Hungary_DanubeTisza_LSarmation_EHun, Hungary_Hun, Hungary_Transtisza_LSarmation_EHun, Hungary_Transtisza_Roman_Sarmation, Ukraine_IA_WesternScythian, Ukraine_Scythian

Kazakhstan Sarmatian (Kazakhstan_Sarmatian_IA):

Northwest Kazakhstan (between Caspian Sea and Ural Mountains)

Seven individuals dated 3rd C. BCE - 3rd C. CE.

Initially included in steppe_posthunnic group but later separated due to specific role in population processes.
